# Endogenous Retrovirus EAV-HP Linked to Blue Egg Phenotype in Mapuche Fowl

**DOI:** 10.1371/journal.pone.0071393

**Published:** 2013-08-19

**Authors:** David Wragg, Joram M. Mwacharo, José A. Alcalde, Chen Wang, Jian-Lin Han, Jaime Gongora, David Gourichon, Michèle Tixier-Boichard, Olivier Hanotte

**Affiliations:** 1 Centre for Genetics and Genomics, School of Biology, University of Nottingham, University Park, Nottingham, United Kingdom; 2 Pontificia Universidad Catolica de Chile, Facultad de Agronomia e Ingenieria Forestal, Santiago, Chile; 3 CAAS-ILRI Joint Laboratory on Livestock and Forage Genetic Resources, Institute of Animal Science, Chinese Academy of Agricultural Sciences (CAAS), Beijing, China; 4 International Livestock Research Institute (ILRI), Nairobi, Kenya; 5 The University of Sydney, Faculty of Veterinary Science, Sydney, New South Wales, Australia; 6 Institut National de la Recherche Agronomique, UE1295 Poultry Experimental Platform of Tours, Nouzilly, France; 7 Institut National de la Recherche Agronomique, AgroParisTech, UMR1313 Animal Genetics and Integrative Biology, Jouy-en-Josas, France; National Institute of Allergy and Infectious Diseases, United States of America

## Abstract

Oocyan or blue/green eggshell colour is an autosomal dominant trait found in native chickens (Mapuche fowl) of Chile and in some of their descendants in European and North American modern breeds. We report here the identification of an endogenous avian retroviral (EAV-HP) insertion in oocyan Mapuche fowl and European breeds. Sequencing data reveals 100% retroviral identity between the Mapuche and European insertions. Quantitative real-time PCR analysis of European oocyan chicken indicates over-expression of the *SLCO1B3* gene (*P*<0.05) in the shell gland and oviduct. Predicted transcription factor binding sites in the long terminal repeats (LTR) indicate *AhR/Ar*, a modulator of oestrogen, as a possible promoter/enhancer leading to reproductive tissue-specific over-expression of the *SLCO1B3* gene. Analysis of all jungle fowl species *Gallus sp.* supports the retroviral insertion to be a post-domestication event, while identical LTR sequences within domestic chickens are in agreement with a recent *de novo* mutation.

## Introduction

The ancestral avian eggshell is believed to be both white and immaculate [1], whilst the eggshells of modern birds are diverse in both colour and maculation. The diversity in colour can be attributed to pyrroles [2], and it has been confirmed across a range of avian species that the principal eggshell pigments are protoporphyrin and biliverdin [2]–[4]. Protoporphyrin gives rise to brown eggs, whereas the bile pigment biliverdin gives rise to blue or green eggs in the presence of protoporphyrin; white eggs, however, may contain low concentrations of one or both detectable pigments, or none at all [3]. A study in the pigmentation of colourful eggshells of extinct Dinornithidae [4] expands on this conclusion by proposing that pyrrole eggshell pigments are both ancient in origin and highly conserved.

Several hypotheses have been proposed to account for the diversity of eggshell pigmentation. These range from the conspicuous, crypsis and recognition [1], to the less conspicuous such as structural integrity [5] immunocompetence [6], and luminance – thermo-regulation, UV-B protection, photo-acceleration, lateralization, circadian rhythm, photo-reactivation, and antimicrobial defence [7]. A detailed assessment [8] of museum eggshells from 49 British bird species (neoaves) identified protoporphyrin concentration to be associated with species that lay maculated eggs, finding it to be high in both ground- and cavity-nesting species. Protoporphyrin may have a role independent of signalling, and could possibly be involved in microbial defence [8]. In contrast, biliverdin has been associated with non-cavity nesting habits and an increased propensity for bi-parental provisioning [8]. It is more likely than not, that a combination of different ecological and evolutionary pressures have culminated in the diversity of eggshell pigmentation found in modern birds with varying life histories [9].

Oocyan is an autosomal dominant trait in chicken resulting from an accumulation of biliverdin in the eggshell, leading to blue/green shelled eggs [10]. It is found among the native domestic chickens of Chile, known as Mapuche fowl, and among some chicken breeds of Asia as for instance in the Dongxiang breed [11]. The Mapuche fowl [10] is the designation of the native Chilean fowl associated with the Mapuche people known as the ‘Araucanos’ by the Spanish. These fowl include the rumpless blue/green egg laying ‘kollonca’ and the tailed ear-tufted ‘ketro’ which lays mostly brown eggs. Crossing of the kollonca and ketro gave origin to the tufted rumpless standard of the Araucana breed from North America and Europe during the 20th century [12]. Two hypotheses may be proposed concerning the presence of oocyan chicken in South America: (i) an introduction from outside the continent, or (ii) independent *de novo* origin in South American chicken. Following Castello [12], blue eggs were present in Chilean Araucana chickens (herein refered to as Mapuche fowl) in the 19th century, as witnessed by Dr Ruben Bustos during the Pacific War (1879–84). Green (iridescent) egg laying chicken (Dongxiang; [13]) might have been present for more than 500 years in today's People's Republic of China (herein referred to as China).

Recently Wang *et al*. [14] identified an endogenous avian retroviral (EAV-HP) insertion in Chinese Dongxiang chicken associated with the over-expression of a solute carrier, *SLCO1B3*, proposing it to be the causative mutation of the oocyan phenotype in the breed. They reveal the same retroviral insertion and site to be present in another Chinese breed (Lushi), but a different insertion site in North American Araucana believed to be of South American descent. They suggest that these two insertions represent parallel evolution of the oocyan phenotype in the American and Asian continents, but do not provide any evidence of enhanced expression of *SLCO1B3* in native fowl from the South American continent.

Here, we report the sequencing of the 300 kb interval containing the oocyan locus that we previously mapped in Mapuche fowl and European chicken breeds [15]. We identify that an EAV-HP insertion is likely responsible for the oocyan phenotype. Moreover, we found conserved EAV-HP integration sites and sequences in South American and European oocyan chickens, distinct from those of the Asian chicken [14]. We show that this insertion enhances the expression of the neighbouring solute carrier *SLCO1B3* in the shell gland and oviduct of European oocyan chickens and that *HMOX1,* a previous candidate gene, is not over-expressed [16]. Genetic screening in domestic chicken and wild jungle fowl suggests the EAV-HP insertion is a *de novo* mutation in domestic chicken. Our results support the parallel, post-domestication, integration of an endogenous retrovirus leading to the oocyan phenotype in South America and Asian chickens [14], providing no support for an introduction of the phenotype to South America from Asia or *vice versa*. Moreover, they indicate a likely recent origin for the mutation on the South American continent.

## Results

### Targeted re-sequencing hints at an EAV-HP integration in oocyan mapping interval

**Figure 1:**
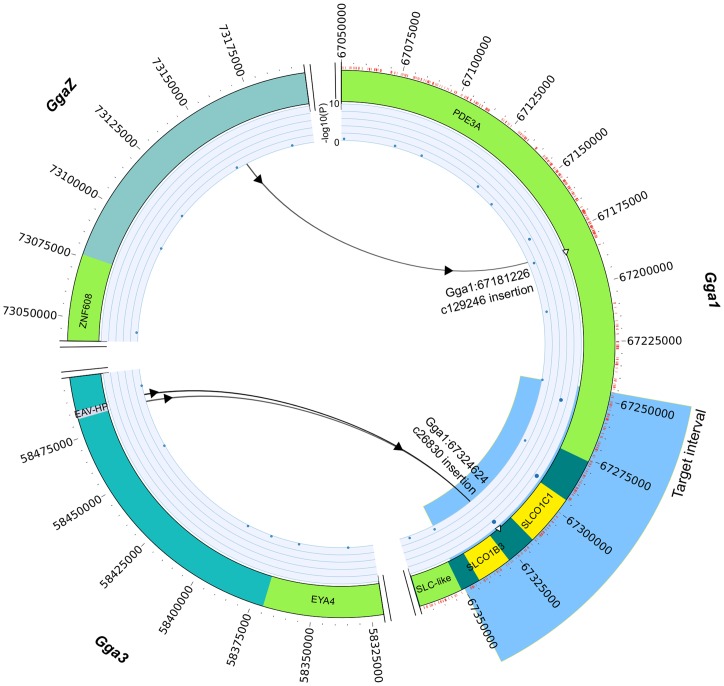
Circos plot illustrating oocyan mapping, target interval, SNPs and structural variants. SNPs indicated as red tick marks on outside of ideogram; GWAS mapping indicated on inner track; structural variants (predicted translocations) marked as arrows indicating sources and destination chromosomes; genes in green; candidate genes in yellow; EAV-HP on *Gga3* indicated in light blue.

Target enrichment and sequence capture in 9 chickens (3 oocyan, 6 non-oocyan; Table S1) including 5 native Mapuche fowl, result in the identification of 210 SNPs unique to oocyan chickens within the target mapping interval (*Gga1*:67,246,039–67,350,164) (Figure 1). Variant effect prediction indicates that none of the SNPs results in non-synonymous mutation (Table 1). Structural variant (SV) analyses identify two SVs (c26830 and c129246) common to the oocyan chickens. SV c129246 is located in the *PDE3A* gene, ∼65 kb upstream of the target interval; whilst c26830 is within the target interval, in the intergenic space between the two candidate genes, *SLCO1C1* and *SLCO1B3* (Figure 1) [15]. The assembled orphaned reads of c26830 reveal a sequence homology with the long-terminal repeats (LTRs) of the EAV-HP present on chromosome 3 (*Gga3*:58,482,495–58,485,879) of the chicken reference genome (galGal3, May 2006), indicating c26830 to be a possible retroviral insertion rather than a translocation.

**Table 1:** Summary of Ensembl Variant Effect Predictor results for oocyan-unique SNPs in captured region.

Variant	Intergenic	*PDE3A*	*SLCO1C1*	*SLCO1B3*	*LOC418189*
Intergenic	27	–	–	–	–
Upstream gene	–	0	30	6	0
Intron	–	166	61	12	0
Missense	–	2	0	0	0
Synonymous	–	2	4	2	0
Splice region (intron)	–	2	1	2	0
Splice region (non-synonymous)	–	0	2	0	0
Downstream gene	–	22	12	9	5

### PCR confirms retroviral nature of the insertion

Long-range PCR confirms the insertion to be oocyan specific, and ∼4.5 kb in size (Figure S1). Multiplex PCR screening of a diverse range of domestic chickens of known phenotype produces the expected amplicon size, 167 bp for oocyan (n = 45) and 364 bp for non-oocyan (n = 33) birds. Genotype frequencies are presented in Table 2. A 364 bp product was amplified in green (*G. varius*), grey (*G. sonneratii*), and Sri Lankan (*G. lafayettii*) jungle fowl, as well as three red jungle fowl subspecies (*G. g. bankiva*, *G. g. gallus*, and *G. g. spadiceus*), none of which lays blue eggs, indicating the conserved length of the genomic region amplified and absence of the insertion (Table 2). The multiplex PCR was used to genotype 51 chickens of unknown egg phenotype from a Dongxiang population in which oocyan segregates. In these chickens the amplicon associated with the insertion is found to be slightly longer by about ∼20 bp compared to South American/European oocyan chickens (Figure S2).

**Table 2:** Frequency of genotypes observed by multiplex PCR.

Breed	N	Phenotype	O*N	O*LC/O*N	O*LC
Araucana	10	Blue	0.00	0.60	0.40
Cream Legbar	2	Blue	0.00	0.00	1.00
Crèvecoeur	1	Non-Blue	1.00	0.00	0.00
Dongxiang	51	Unknown	0.16	0.68	0.16
Mapuche Fowl	23	Non-Blue	1.00	0.00	0.00
Mapuche Fowl	31	Blue	0.00	0.71	0.29
Moss	2	Blue	0.00	1.00	0.00
Rhode Island Red	4	Non-Blue	1.00	0.00	0.00
White Leghorn	4	Non-Blue	1.00	0.00	0.00
White Star	1	Non-Blue	1.00	0.00	0.00
*G. g. bankiva*	2	Non-Blue	1.00	0.00	0.00
*G. g. gallus*	2	Non-Blue	1.00	0.00	0.00
*G. g. spadiceus*	4	Non-Blue	1.00	0.00	0.00
*G. lafayettii*	2	Non-Blue	1.00	0.00	0.00
*G. sonneratii*	2	Non-Blue	1.00	0.00	0.00
*G. varius*	2	Non-Blue	1.00	0.00	0.00

### Sequencing confirms the insertion to be EAV-HP integration

**Figure 2:**
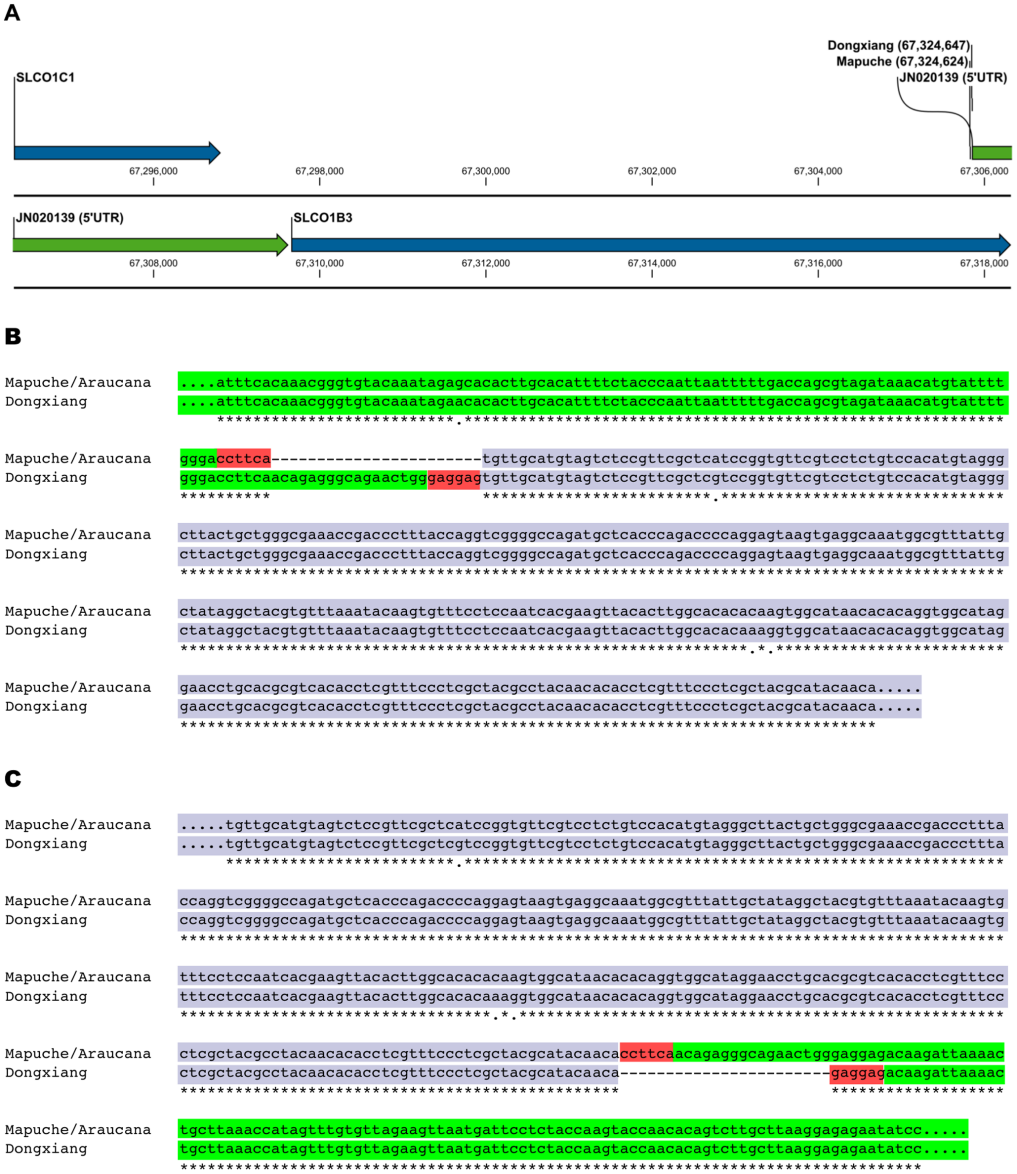
Partial schematic diagram of chromosome 1 illustrating EAV-HP integration sites, and LTR sequence alignment in Mapuche/Araucana and Dongxiang chickens. Complete sequences of the EAV-HP insertions reveal independent integration sites in the Mapuche/Araucana and Dongxiang chickens (a), at the beginning of the 5′ untranslated region (UTR; *Gga1*:67,324,643–67,328,415) of a *SLCO1B3* transcript (GenBank accession no: JN020139). Forward strand multiple sequence alignment of the host sequence upstream (b) and downstream (c) of each integration site is highlighted in green, with EAV-HP LTR sequence highlighted in blue, and TSDs highlighted in red.

The complete insertion was Sanger sequenced by primer-walking in three homozygote oocyan chickens, one each from South America (Chile, Mapuche fowl), Europe (France, Araucana), and Asia (China, Dongxiang). This confirms the insertion to be an EAV-HP retrovirus integrated in opposite orientation to the solute carrier *SLCO1B3* in all three birds. Identical host integration sites (*Gga1*:67,324,624) and DNA sequences (4,243 bp) are found for the South American and European chickens. The EAV-HP sequence of the oocyan Dongxiang chicken is 98% identical to the Mapuche/Araucana, however, the host integration site is different at *Gga1*:67,324,647 (Figure 2). A single base pair mutation (*Gga1*:67,324,554, G instead of A) is found in the 96 bp homologous host sequence upstream of the Mapuche/Araucana retroviral integration site, whilst the Dongxiang shared the reference genome ‘A’ allele (Figure 2b). No polymorphism was observed in the 100 bp homologous host sequence downstream of the retroviral integration site (Figure 2c) in any of the sequences.

The target-site duplication (TSD) is different in the Mapuche/Araucana (3′-CCTTCA-5′) and Dongxiang (3′-GAGGAG-5′) chicken. The opposing LTRs within a given sequence are identical for their 313 bp length, although a pair-wise comparison reveals a 1% sequence divergence (3 SNPs) between the Mapuche/Araucana and Dongxiang LTR sequences (Figure 2b). GeneMark predicts a single protein yielding 96% similarity when comparing the Mapuche/Araucana to the Dongxiang sequences, whilst analysis of the protein sequences in Pfam identifies matches for *gag* and *env* domain families but none for the *pol* domain. Annotated DNA sequences for the Mapuche/Araucana and Dongxiang chickens detailing the LTR sequences including U3 domain transcription factor binding sites, Pfam domains, and protein sequences are provided in the supplementary information (Sequence S1 and S2). Aligning our EAV-HP Dongxiang sequence to that of Wang *et al*. ([14]; GenBank accession no: JF837512) identified 5 polymorphisms (C940G, T943-, G944-, G945-, and A2544C) resulting in a sequence divergence of 0.001%; the alignment is also included in the supplementary information (Sequence S3).

**Tissue-specific over-expression of** ***SLCO1B3*** **in presence of the EAV-HP integration**

**Figure 3:**
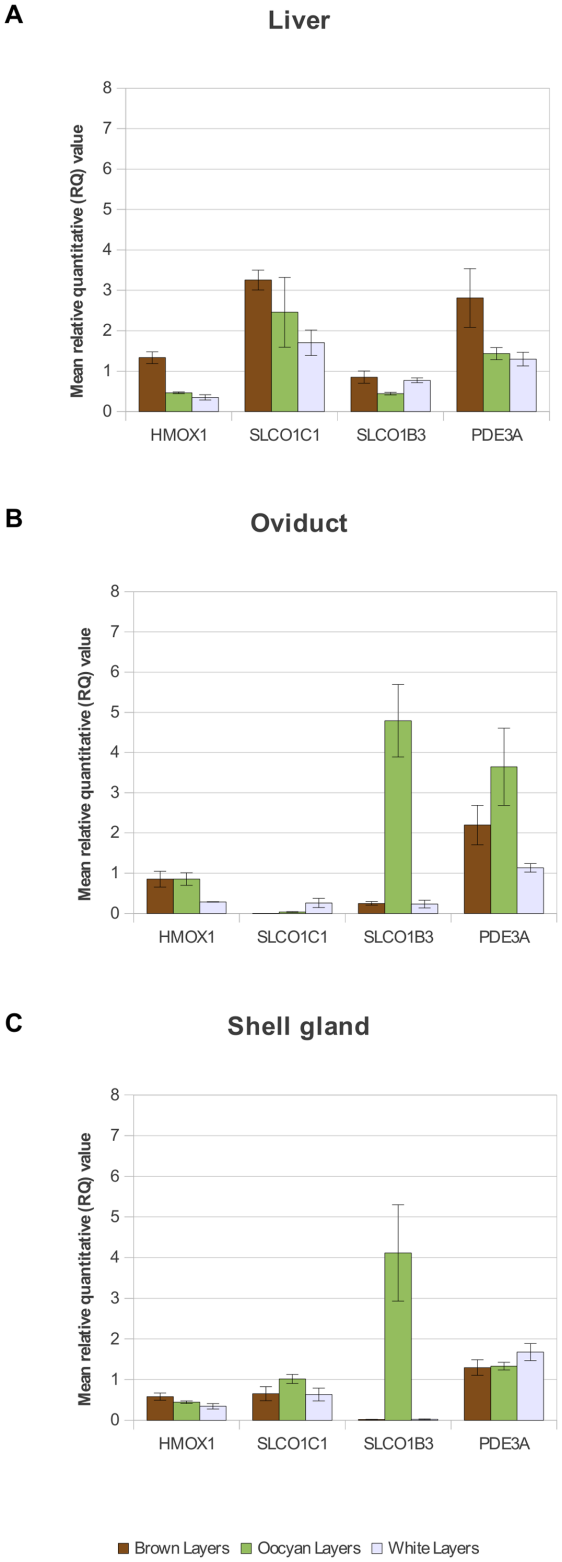
Gene expression results. qRT-PCR results of European chickens laying oocyan, brown and white eggs for *SLCO1C1*, *SLCO1B3*, *HMOX1* and *PDE3A* in the liver, shell gland and oviduct. Error bars indicate standard error of the mean. All samples tested were recorded as having a calcified egg in the shell gland post-slaughter.

To assess the possible impact of the retroviral insertion on the expression of the solute carriers, qRT-PCR was performed using cDNA obtained from the shell gland, oviduct and liver homogenates harvested from oocyan, white and brown egg layers. Multiplex PCR confirmed the absence or presence of the oocyan EAV-HP insertion in relevant samples selected for qRT-PCR. Prior to the experiment, we analysed the stability of expression of the *UB* and *G6PDH* house-keeping genes in all three tissue types. The analysis revealed the expression of *UB* to be consistent in all three tissues while that of *G6PDH* was variable, as has also recently been observed by Lin and Redies [17]. Based on these results, we used the *UB* gene as the internal control for normalization of the levels of expression of the four target genes (*SLCO1C1*, *SLCO1B3*, *HMOX1*, and *PDE3A*). The average relative quantitative (RQ) values are presented in Table S2 together with significance values following Student's *t*-test. For the qRT-PCR runs, each target gene was analysed in triplicate in each tissue. Significant (*P*<0.05) over-expression of *SLCO1B3* is observed in the shell gland (∼19 fold increase) and oviduct (∼180 fold increase) of oocyan chickens (Figure 3). The EAV-HP integration is in opposite orientation to its solute carrier neighbours, and the over-expression of *SLCO1B3* suggests the retrovirus to be acting as an enhancer insertion. We found no significant association between blue eggs and the over-expression of *HMOX1* (Figure 3, Table S2).

## Discussion

The close genomic proximity of the EAV-HP integration to *SLCO1B3*, its unique presence in oocyan chickens and the tissue-specific over-expression of the solute carrier, known to transport bile salts such as biliverdin, strongly supports the retroviral insertion as the causative mutation of the oocyan phenotype in Mapuche fowl and their modern (European and North American) descendants. Taken together with the independent discovery of the EAV-HP integrations by Wang *et al*. [14], in Chinese Dongxiang and Lushi chickens, as well as the evidence of *SLCO1B3* over-expression in oviduct tissue in Dongxiang chickens, the results complement one-another. With the knowledge of distinct genomic insertion sites, the results clearly indicate the independent acquisition of the oocyan phenotype in native Asian and South American chickens.

The origin of chickens in South America is a subject of debate, and genetic links to Chinese chicken have been suggested [18]. In this respect, the presence of the oocyan phenotype on continental Asia and South America could have been interpreted as evidence of ancient seafaring links between the two continents. The findings by Wang *et al.* [14] and this study on the independent acquisition of the phenotype, indicate that the oocyan phenotype is offering no insight on the origin of South American chickens.

Historical evidence for the oocyan phenotype indicates that it has been present since at least 500 years ago in the Dongxiang chicken in Asia [13] and the late 19th century with a large geographic distribution in South America [12]. The lack of divergence in the LTR sequences of the EAV-HP insertions both within the Dongxiang breed and the Mapuche fowl supports a relatively recent integration event on both continents.

The EAV-HP integration site of the oocyan Mapuche fowl, the French Araucana, the Moss breed from Spain, and the Araucana and Cream Legbar chickens from the UK are identical and in agreement with historical accounts that the modern European oocyan breeds derive from Mapuche fowl. Similarly the genome integration site of the Mapuche fowl was identical to the North American Araucana [14], again confirming the origin of this population. These findings therefore suggest that the presence of blue eggs in Mapuche fowl, modern European and American breeds, did not involve birds from Asia despite suggestions of their travels with Dutch pirates [10], and that the oocyan Asian genotype has remained confined to China. This might be considered surprising given that many modern breeds derive from Asian lineages – for instance the Silkie, Brahma, Cochin and Croad Langshan to name a few, are now commonly found throughout Europe and North America.

None of the jungle fowl species described (*Gallus gallus*, *G. sonneratii*, *G. lafayettii*, and *G. varius*) are known to lay blue or green eggs [19]. However, the presence of EAV-HP has previously been found in the red jungle fowl *G. gallus*, and is likely to have been acquired post separation of *Gallus* from other galliform species due to its absence in the more distant turkey and quail genomes [20]. We verified the absence of the oocyan-specific EAV-HP insertion in jungle fowl species by PCR, demonstrating that this insertion is likely to have occurred post-domestication of *G. gallus*. However, the absence of sequence divergence in the opposing LTRs of the EAV-HP insertion within any of the individual chickens sequenced prevents an estimation of the date of the integration other than to suggest that it is recent.

The EAV-HP retrovirus lacks a *pol* domain, rendering it self-replication defective [20], and so it would likely require an infecting virus acting in *trans* for it to proliferate. It is entirely possible that an exogenous virus might facilitate the proliferation of EAV-HP within the genome as has been demonstrated previously for the Rous Sarcoma virus (RSV; [21]). The close genomic proximity (23 bp) of the different insertion sites, observed in Mapuche fowl and Dongxiang chicken, suggests a possible integration site preference for EAV-HP, as has been suggested for several retroviruses [22]. Active ongoing insertion and segregation of EAV-HP in chicken populations have been shown with a typical prevalence of 10 to 15 copies per genome [23], [24]. Such EAV-HP genome dynamism is thought to play a recombinant role in the emergence of exogenous avian retrovirus ALV-J due to a uniquely similar *env* sequence with the ALV-J prototype HPRS-103 [23]. An intact EAV-HP, including *pol* gene, has previously been identified in *G. sonneratii* [25], and EAV-HP proviruses with intact *pol* and *env* genes have also been found in domestic chicken [23]. Borisenko [26] found the EAV-HP *pol* gene to be more closely related to that of RSV than of other retroviridae. The expressed EAV-HP transcripts in some lines [23], [27] and *pol* sequence homology with RSV support therefore the possibility that a helper virus might be contributing to the continued segregation of EAV-HP.

Avian retroviral LTRs have been extensively studied [28]. They are typically characterized by three domains – U3, R and U5, which are mirrored in the LTRs at opposing ends of the retrovirus. The U3 region accommodates binding sites for cellular proteins that promote transcription initiation, and which are capable of activating or enhancing the expression of neighbouring genes, typically when the retrovirus is in opposite orientation to the affected gene (e.g. as in the case of human salivary amylase genes [29], [30]). A TFSEARCH of the U3 region of the LTR sequences described here identifies a number of predicted transcription factor binding sites that might stimulate the transcription of neighbouring genes. One of these binding sites, *AhR/Ar* (score 85.4), has been linked to oestrogen metabolism and oxidative stress [31], [32], and has also been shown to increase solute carrier expression in the liver of mice [33]. The oocyan Dongxiang *SLCO1B3* transcript (GenBank accession no: JN381032) commences 2 bp after the predicted *AhR/Ar* transcription factor binding site. The results of our analyses therefore suggests that the EAV-HP insertion might enhance the expression of *SLCO1B3* in a reproductive tissue-specific manner. This is possibly due to promoter and/or enhancer sequences in the LTRs, such as *AhR/Ar* – a mediator of oestrogen regulation. Indeed, several studies have identified genes expressed in the chicken oviduct as being under the regulation of oestrogen [34]–[36]. We suspect that the over-expression in the shell gland of *SLCO1B3*, for which biliverdin is a substrate, might increase the accumulation of biliverdin in the shell gland during shell matrix formation, giving rise to blue eggs. However, in absence of full transcriptome data we cannot fully exclude the possibility of other long-range *cis*-regulatory effects of the EAV-HP insertion, or a possible role for any of the SNPs identified in the oocyan mapping interval on the expression of the phenotype.

Our findings address an important question: to what extent are endogenous retroviruses (ERVs) shaping phenotypic diversity in birds? It is not the first reported instance of an ERV insertion affecting a chicken's phenotype. An insertion of avian leukosis virus (ALV) in intron 4 of the tyrosinase gene results in the recessive white plumage of some chicken breeds [37]. Furthermore, late-feathering has been associated with endogenous virus 21 [38] and henny-feathering has been linked to the promoter activity of a retroviral LTR [39]. It should be noted that the absence of an ERV integration in the duck for the homologous region identified for oocyan in chickens [14] does not discount the possibility that a similar mechanism is in place in this species. There are several solute carriers with an organic anion transporting role [40], and the possibility of an endogenous retrovirus influencing a different solute carrier to *SLCO1B3*, resulting in blue eggs cannot be excluded, calling for further investigation.

The abundance of ERVs identified in three neognaths [41] indicates a potential reservoir of retroviridae for shaping phenotypic diversity across birds. Bolissety *et al*. [41] suggest that prehistoric birds were a melting pot for ERVs, and that avian retroviruses have evolved independently from other retroviruses over the last 150 million years. They found a random distribution of ERV integrations in the chicken genome, with 25% near to transcription units and many ERVs translated, some in a tissue-specific manner. A similar distribution of integration sites was observed in the zebra finch *Taeniopygia guttata*, which has more than double the ERV burden compared to chicken [41].

This ERV reservoir together with the capacity of ERVs for horizontal transfer highlights the possibility that ERVs might influence egg colour more generally in birds. For example species like house sparrow *Passer domesticus*, European cuckoo *Cuculus canorus*, and common guillemot *Uria aalge* are all laying blue eggs as well as non-blue eggs within a single population. In this context, it is also interesting to remember that brown egg colours of various intensities are found across different bird species including chicken, such a phenotype could be influenced by the presence of multiple retroviral insertions in agreement with the polygenic control of the trait [5]. Our findings are therefore providing a new entry point to investigate the genetic control of egg colour polymorphism within and across these species.

Whether the blue egg offers an increased propensity for bi-parental provisioning, confers a luminary-related advantage, anti-microbial properties, or offers some degree of crypsis remains to be seen. At least in the case of the chicken, where it has likely been the subject of artificial selection, the oocyan EAV-HP integration will remain in the genome for as long as man continues to take a shine to blue eggs.

## Materials and Methods

### Ethics statement

All the chickens at the INRA experimental farm were produced, fed and sacrificed according to French regulations in 2007, which did not require the approval by an ethical committee at that time, but the authorization of the facility and the researchers. The farm was, and still is, registered by the Ministry of Agriculture with the license number B37–175–1 for animal experimentation. The experiment was realised under authorization 37–002 delivered to D. Gourichon and authorization 2369 delivered to M. Tixier-Boichard. Animal procedures were approved by the Departmental Direction of Veterinary Services of Indre-et-Loire. Before tissue sampling, animals were sacrificed by cervical dislocation followed by bleeding.

### Sample collection

Details of all samples used for this study are provided in Table S3. In summary these included 24 samples from Europe (seven breeds), 51 from Asia (Chinese Dongxiang breed), 54 indigenous chickens from South America (Chilean Mapuche fowl) and 14 jungle fowl (*Gallus sp*.). Details concerning DNA isolation and processing of tissues for RNA isolation are included in the supplementary information (Methods S1a).

### RNA preparation

Total RNA was isolated from the liver, shell gland and oviduct of four individuals each of oocyan, brown, and white egg laying chickens using TRIzol® Reagent (Ambion®, Life Technologies Ltd) following the manufacturers recommended approach. All samples were recorded as having a calcified egg in the shell gland. The concentration and purity of all RNA samples was determined with a Nano-Drop® ND-1000 UV-Vis Spectrophotometer (Nano-Drop Technologies). Samples were aliquoted into 50 µl volumes at a concentration of 10 µg and stored at −80°C until use. Prior to the qRT-PCR amplification, aliquoted RNA (10 µg in 50 µl reaction volumes) was treated with TURBO Dnase I (Ambion®, Life Technologies Ltd) to remove any trace genomic DNA arising from the isolation procedure. The High Capacity RNA-to-cDNA Kit (Invitrogen) was used to synthesize *in vitro* cDNA from total RNA following the manufacturers recommended protocol. Both positive and negative reactions were carried out to ensure specificity of template amplification and non-contamination. The oocyan genotypes of the birds selected for gene expression studies were confirmed by diagnostic PCR.

### Target enrichment and sequence capture

Target enrichment was performed using Agilent's SureSelect^XT^ Custom Target Enrichment Kit (Agilent Technologies Inc). Baits were designed using Agilent's eArray software (https://earray.chem.agilent.com/earray) to capture the region previously mapped for oocyan (*Gga1*:67,051,487–67,364,512) in three oocyan (Araucana, n = 2; Mapuche fowl, n = 1) and six non-oocyan chickens (Crèvecoeur, n = 1; Mapuche fowl, n = 4; White Star n = 1). Paired-end (2×100 bp) sequencing was performed using Illumina's GAIIx platform. Reads were mapped using BWA [42] to galGal3 (May, 2006), with read trimming quality at 20 (-q 20), allowing gaps up to 5 bp (-e 5) and disallowing long deletions within 5 bp of the 3′-end of the reads (-d 5). Local realignment was performed using default parameters of GATK [43], and duplicates removed using Picard (http://picard.sourceforge.net). BAM files from the target-enrichment sequence-capture for the nine chickens sequenced have been uploaded to NCBI (SRA accession no: SRA067224).

**Variant discovery**

SNPs were called using GATK's Unified Genotyper [44] employing non-default values for down-sample coverage (1000), minimum base quality score (9) and minimum indel count (2). SNPs were filtered by comparing oocyan to non-oocyan chickens and the reference allele. Oocyan-unique SNPs were filtered against both the Ensembl (http://www.ensembl.org/info/data/ftp) and BGI (http://chicken.genomics.org.cn/chicken/) SNP databases, and the functional consequences of novel SNPs were summarized using Ensembl's Variant Effect Predictor [45] (Table 1). Structural variants (SVs) were called using GASV 2.0 [46] and filtered to produce a set of SVs unique to oocyan samples and within the mapping interval (*Gga1*:67,051,487–67,364,512). Reads spanning the SV boundaries were assembled into contigs and BLAT against the chicken genome (galGal3) to resolve the SV break-point to base pair resolution and to identify partial sequences of the insertion. A detailed account of SV analysis is provided in the supplementary information (Methods S1b).

### Primer design and PCR

All primers used in the study are provided in Tables S4 and S5, and all PCR protocols are described in detail in the supplementary information (Methods S1c). The quantitative real-time PCR (qRT-PCR) primers were all intron-spanning with the exception of *SLCO1B3* which included a primer spanning an exon-exon junction. The *UB* and *G6PDH* house-keeping genes [47] were tested for their suitability as internal controls for normalization by 1.2% agarose gel electrophoresis following their amplifications in PCR using cDNA from the three tissues. The expression of the *UB* gene was much more consistent across all tissues and was thus used as the internal control. The target genes tested included *SLCO1C1*, *SLCO1B3*, *HMOX1* whose over-expression has been shown to be associated with oocyan in Dongxiang chickens [16], and also *PDE3A* which lies upstream of *SLCO1C1* and hosts a possible SV (c129246). Data were collected and analysed using the 7500 Fast Real-Time PCR Systems v2.0.6 software (Life Technologies) and the RQ values for each tissue and target were exported to Excel®. An average value was calculated and the relative RQ plots were generated for each target for oocyan, brown and white egg layers respectively. Differences in expression levels for each target between oocyan and non-oocyan chickens were tested for their significance using Student's *t*-test.

The size of the c26830 retroviral DNA insertion was identified by long-range touch-down PCR. Primers spanning the insertion breakpoint on *Gga1* were designed to amplify a 349 bp product in non-oocyan chickens. A PCR amplification band was observed at ∼4.5 kb in oocyan samples extracted from blood or tissue, but was inconsistent in oocyan samples extracted from blood on FTA Classic cards – these samples were later confirmed by multiplex PCR (described below) to contain the retroviral insertion. The failure to consistently amplify the 4.5 kb fragment from the FTA samples might be explained by degraded DNA, or high amounts of inhibitor carry-over following DNA extraction. The c26830 insertion was Sanger sequenced in three oocyan homozygotes, one each of the Dongxiang breed, European Araucana breed and a Mapuche fowl, by primer-walking using the c26830 long-range PCR product as the template. FASTA sequences have been submitted to NCBI GenBank for the Mapuche/Araucana and Dongxiang EAV-HP insertions (GenBank accession no: KC632577 and KC632578).

Primers were designed to perform a multiplex PCR to screen a large number of samples for the retroviral insertion. One of the reverse primers (Multiplex PCR R2) is in the LTR sequence of EAV-HP which, with the forward primer, amplifies a 167 bp product (190 bp in Dongxiang) in the presence of the retroviral insertion. Whilst the other reverse primer (Multiplex PCR R1) with the forward primer spans the insertion breakpoint on *Gga1* and amplifies a 364 bp product in the absence of the retroviral insertion. In total, 143 samples were screened (14 jungle fowl, 51 Dongxiang chickens, 54 Mapuche fowl, and 24 individuals of various modern and traditional chicken breeds) (Table S3).

### EAV-HP sequence analysis

A BLAST of the complete c26830 sequence confirmed it to be the EAV-HP retrovirus. The heuristic approach for gene prediction [48] for viruses implemented in GeneMark [49] was used to predict genes in the complete c26830 sequence, following which, the predicted protein sequence was analysed for protein families using Pfam [50]. Transcription factor binding sites were predicted for the LTR U3 domains of each sequence using TFSEARCH and the vertebrate TRANSFAC database [51].

## Supporting Information

Figure S1 Long-range PCR spanning the c26830 insertion.
(PDF)

Figure S2 Multiplex PCR reveals a different product size in Dongxiang chicken.
(PDF)

Table S1 Target-enrichment sequence capture mapping summary.
(PDF)

Table S2 Average relative quantitative (RQ) values per sample per tissue following qRT-PCR.
(PDF)

Table S3 Summary of samples used in the study.
(PDF)

Table S4 Primers used in qRT-PCR.
(PDF)

Table S5 Primers used in long-range and multiplex PCRs.
(PDF)

Methods S1 Supplementary information on (a) sampling, (b) structural variant discovery, and (c) PCR cycling profiles.
(PDF)

Sequence S1 Annotated sequence for Mapuche/Araucana oocyan homozygote.
(PDF)

Sequence S2 Annotated sequence for Dongxiang oocyan homozygote.
(PDF)

Sequence S3 Sequence alignment of Dongxiang sequence to GenBank accession no: JF837512.
(PDF)
