# Correction: Endogenous Retrovirus EAV-HP Linked to Blue Egg Phenotype in Mapuche Fowl

**DOI:** 10.1371/annotation/2727de85-899f-4104-955d-1c5f3c0d3319

**Published:** 2013-08-29

**Authors:** David Wragg, Joram M. Mwacharo, José A. Alcalde, Chen Wang, Jian-Lin Han, Jaime Gongora, David Gourichon, Michèle Tixier-Boichard, Olivier Hanotte

There was an error in the Funding statement. The correct version of the Funding statement is available below:

Funding for this study was made possible through a BBSRC PhD fellowship to the first author and a BBSRC research grant (BH/H009051/1) to the last author, as well as the Chinese Government contribution to the CAAS-ILRI Joint Laboratory on Livestock and Forage Genetic Resources. The funders had no role in study design, data collection and analysis, decision to publish, or preparation of the manuscript. 

