# Correction: Endogenous Retrovirus EAV-HP Linked to Blue Egg Phenotype in Mapuche Fowl

**DOI:** 10.1371/journal.pone.0116015

**Published:** 2014-12-15

**Authors:** The PLOS ONE Staff

There is an error in the sequence length calculation in the second sentence of the first paragraph of the “Sequencing confirms the insertion to be EAV-HP integration” section in the Results. The correct sentence is: Identical host integration sites (/Gga1/:67,324,624) and DNA sequences (4,250 bp) are found for the South American and European chickens.

There is an error in the last sentence of the second paragraph of the “Sequencing confirms the insertion to be EAV-HP integration” section in the Results. The value was reported as a fraction, rather than a percentage. The correct sentence is: Aligning our EAV-HP Dongxiang sequence to that of Wang *et al*. ([14]; GenBank accession no: JF837512) identified 5 polymorphisms (C940G, T943-, G944-, G945-, and A2544C) resulting in a sequence divergence of 0.119%; the alignment is also included in the supplementary information (Sequence S3).

## References

[1] Wragg D, Mwacharo JM, Alcalde JA, Wang C, Han J-L, et al (2013) Endogenous Retrovirus EAV-HP Linked to Blue Egg Phenotype in Mapuche Fowl. PLoS ONE 8(8): e71393 doi:10.1371/journal.pone.0071393 23990950 10.1371/journal.pone.0071393 PMC3747184

