# Supplementary material for: Endogenous Retrovirus EAV-HP Linked to Blue Egg Phenotype in Mapuche Fowl
Source: PLoS One. 2013 Aug 19;8(8):e71393. doi: 10.1371/journal.pone.0071393 (PMC3747184; doi:10.1371/journal.pone.0071393)
Supplement: Figure S1 — Long-range PCR spanning the c26830 insertion. (PDF) [file pone.0071393.s001.pdf]

A **B**

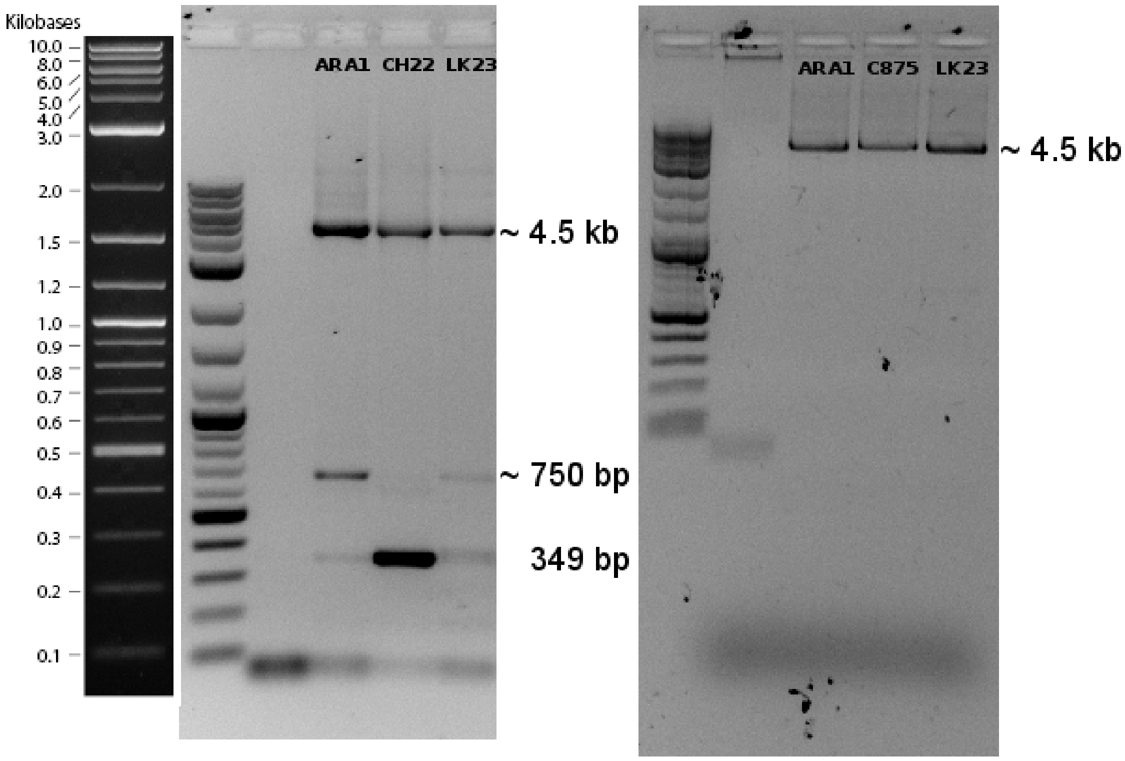

Supplementary Figure S1. Long-range PCR spanning the c26830 insertion

A Long-range PCR of two homozygotes (ARA1, LK23) and a heterozygote (CH22) bird for the oocyan phenotypes shows an amplification band around 4.5 kb. **B** Long-range PCR of three homozygotes (ARA1, C875, LK23) with the touch-down PCR protocol (see Materials and Methods) removing non-specific amplifications around 750 bp observed previously. The precise size of the 349 bp amplification band has been calculated from the reference genome (galGal3) during primer design, whilst the ~ 750 bp and ~ 4.5 kb amplification bands have been estimated from the ladder. PCR products were analyzed by 0.8% agarose gel electrophoresis using a 2-Log DNA ladder (0.1 – 10 kb range) from New England BioLabs (UK) Ltd.
