# Supplementary material for: Endogenous Retrovirus EAV-HP Linked to Blue Egg Phenotype in Mapuche Fowl
Source: PLoS One. 2013 Aug 19;8(8):e71393. doi: 10.1371/journal.pone.0071393 (PMC3747184; doi:10.1371/journal.pone.0071393)
Supplement: Figure S2 — Multiplex PCR reveals a different product size in Dongxiang chicken. (PDF) [file pone.0071393.s002.pdf]

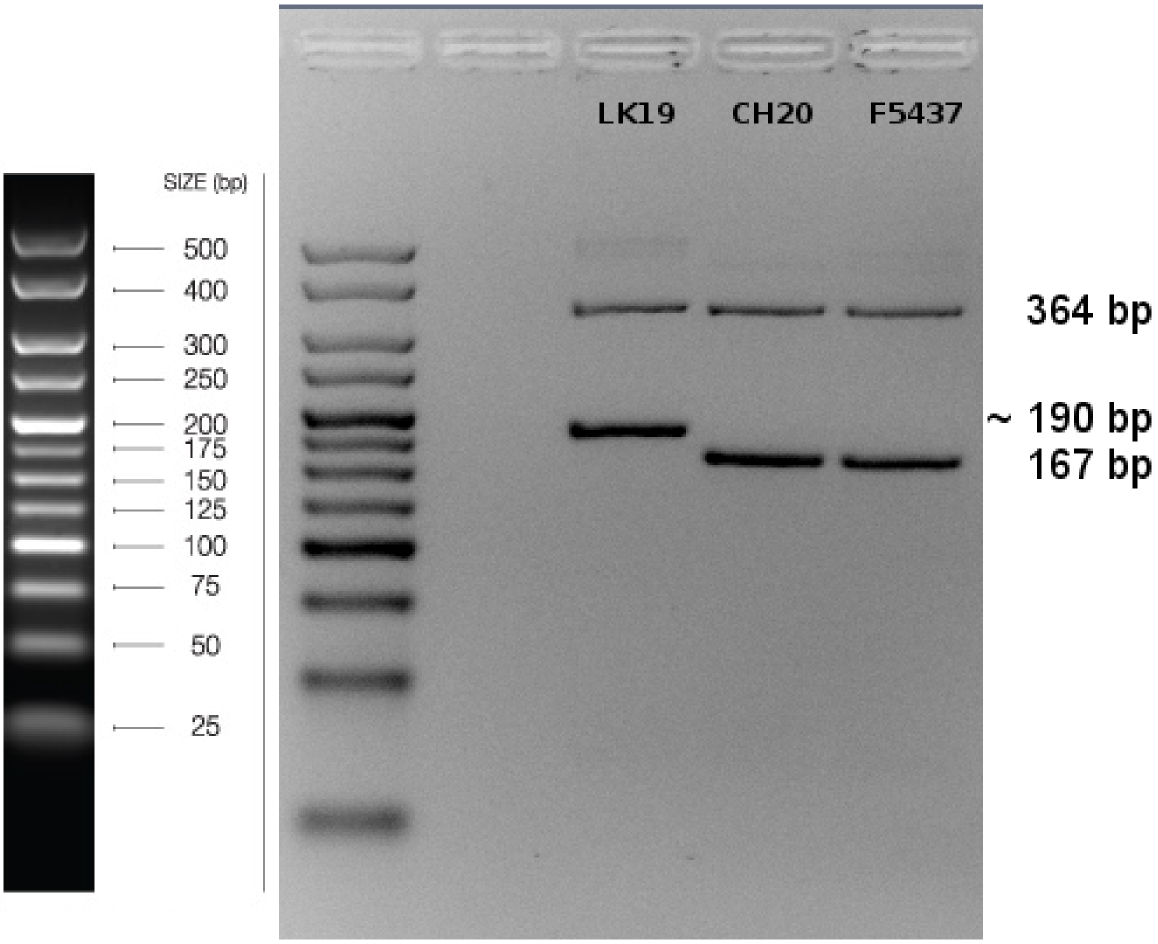

Supplementary Figure S2. Multiplex PCR reveals a different product size in Dongxiang chicken

Multiplex PCR of three samples reveals a slightly larger amplification band (~ 190 bp) in the Dongxiang chicken (LK19) with the EAV-HP specific primer, than that observed in the Mapuche fowl (CH20) and European Araucana (F5437) chickens (167 bp). All three samples also amplify an amplification band at the expected 364 bp for DNA without the EAV-HP insertion with the non-EAV-HP primer indicating them to be heterozygous for the oocyan phenotype. The precise size of the 164 bp and 364 bp amplification bands has been calculated from the reference genome (galGal3) during primer design, whilst the ~ 190 bp amplification band has been estimated from the ladder and was subsequently confirmed through sequencing. PCR products were analyzed by 3.5% agarose gel electrophoresis using HyperLadder V (Bioline Reagents Ltd).
