# Supplementary material for: Endogenous Retrovirus EAV-HP Linked to Blue Egg Phenotype in Mapuche Fowl
Source: PLoS One. 2013 Aug 19;8(8):e71393. doi: 10.1371/journal.pone.0071393 (PMC3747184; doi:10.1371/journal.pone.0071393)
Supplement: Table S1 — Target-enrichment sequence capture mapping summary. (PDF) [file pone.0071393.s003.pdf]

Supplementary Table S1. Target-enrichment sequence capture mapping summary

Breed	Sample ID	Origin	% Reads aligned to galGal3	% Reads aligned to capture regions	Mean depth of coverage
**Oocyan**					
Araucana	ARA1	UK	98.53	82.32	197
Araucana	FR2424	France	98.29	81.05	155
Mapuche fowl	CH20-509	Pirque, Chile	98.31	80.89	145
**Non-oocyan**					
Crevecoeur	CRE10	UK	98.53	82.13	206
Mapuche fowl	CH04-280	Pirque, Chile	98.38	81.62	283
Mapuche fowl	CH11-504	Pirque, Chile	98.30	81.18	166
Mapuche fowl	CH19-832	Villa Cautin, Chile	98.31	80.98	169
Mapuche fowl	CH21-200	Pirque, Chile	98.37	81.13	164
White Star	WST1T1	UK	98.33	81.63	201
