# Supplementary material for: Endogenous Retrovirus EAV-HP Linked to Blue Egg Phenotype in Mapuche Fowl
Source: PLoS One. 2013 Aug 19;8(8):e71393. doi: 10.1371/journal.pone.0071393 (PMC3747184; doi:10.1371/journal.pone.0071393)
Supplement: Table S2 — Average relative quantitative (RQ) values per sample per tissue following qRT-PCR. (PDF) [file pone.0071393.s004.pdf]

Supplementary Table S2. Average relative quantitative (RQ) values per sample per tissue following qRT-PCR

Egg	Oviduct					Shell gland					Liver				
	Sample	SLCO1C1	HMOX1	SLCO1B3	PDE3A	Sample	SLCO1C1	HMOX1	SLCO1B3	PDE3A	Sample	SLCO1C1	HMOX1	SLCO1B3	PDE3A
Brown	BR(OB18)	0.0036	0.8220	0.3792	3.3629	BR(OB18)	1.3514	0.8577	0.0130	1.9351	BR(OB18)	4.0118	1.5761	1.3845	3.1727
	BR(OB18)	0.0036	0.8220	0.3792	3.3629	BR(OB18)	1.3514	0.8577	0.0130	1.9351	BR(OB18)	4.0118	1.5761	1.3845	3.1727
	BR(OB18)	0.0036	0.8220	0.3792	3.3629	BR(OB18)	1.3514	0.8577	0.0130	1.9351	BR(OB18)	4.0118	1.5761	1.3845	3.1727
	BR(OB21)	0.0022	1.5440	0.2841	2.9810	BR(OB21)	0.2937	0.6439	0.0061	1.3373	BR(OB21)	2.3458	0.7631	0.3349	0.1362
	BR(OB21)	0.0022	1.5440	0.2841	2.9810	BR(OB21)	0.2937	0.6439	0.0061	1.3373	BR(OB21)	2.3458	0.7631	0.3349	0.1362
	BR(OB21)	0.0022	1.5440	0.2841	2.9810	BR(OB21)	0.2937	0.6439	0.0061	1.3373	BR(OB21)	2.3458	0.7631	0.3349	0.1362
	BR(OV10)	0.0016	0.1812	0.0839	0.2390	BR(OV10)	0.3187	0.2413	0.0372	0.6158	BR(OV10)	3.4009	1.6598	0.8349	5.1206
	BR(OV10)	0.0016	0.1812	0.0839	0.2390	BR(OV10)	0.3187	0.2413	0.0372	0.6158	BR(OV10)	3.4009	1.6598	0.8349	5.1206
	BR(OV10)	0.0016	0.1812	0.0839	0.2390	BR(OV10)	0.3187	0.2413	0.0372	0.6158	BR(OV10)	3.4009	1.6598	0.8349	5.1206
	**Mean**	**0.0025**	**0.8491**	**0.2491**	**2.1943**	**Mean**	**0.6546**	**0.5810**	**0.0188**	**1.2961**	**Mean**	**3.2528**	**1.3330**	**0.8514**	**2.8098**
	**SD**	**0.0009**	**0.5905**	**0.1305**	**1.4758**	**SD**	**0.5227**	**0.2710**	**0.0141**	**0.5721**	**SD**	**0.7299**	**0.4289**	**0.4547**	**2.1754**
Oocyan	GR(OV05)	0.0557	1.1501	6.6752	3.7257	GR(OV05)	0.8614	0.3803	8.8564	1.6772	GR(OV05)	5.8507	0.5364	0.4157	1.5204
	GR(OV05)	0.0557	1.1501	6.6752	3.7257	GR(OV05)	0.8614	0.3803	8.8564	1.6772	GR(OV05)	5.8507	0.5364	0.4157	1.5204
	GR(OV05)	0.0557	1.1501	6.6752	3.7257	GR(OV05)	0.8614	0.3803	8.8564	1.6772	GR(OV05)	5.8507	0.5364	0.4157	1.5204
	GR(OV08)	0.0446	1.1819	6.5039	6.9340	GR(OV08)	0.7419	0.3821	1.5196	1.2992	GR(OV08)	0.2088	0.3840	0.3575	1.9076
	GR(OV08)	0.0446	1.1819	6.5039	6.9340	GR(OV08)	0.7419	0.3821	1.5196	1.2992	GR(OV08)	0.2088	0.3840	0.3575	1.9076
	GR(OV08)	0.0446	1.1819	6.5039	6.9340	GR(OV08)	0.7419	0.3821	1.5196	1.2992	GR(OV08)	0.2088	0.3840	0.3575	1.9076
	GR(OB25)	0.0028	0.2273	1.1928	0.2791	GR(OV14)	1.4472	0.5724	1.9720	1.0173	GR(OV14)	1.3135	0.4703	0.5562	0.8748
	GR(OB25)	0.0028	0.2273	1.1928	0.2791	GR(OV14)	1.4472	0.5724	1.9720	1.0173	GR(OV14)	1.3135	0.4703	0.5562	0.8748
	GR(OB25)	0.0028	0.2273	1.1928	0.2791	GR(OV14)	1.4472	0.5724	1.9720	1.0173	GR(OV14)	1.3135	0.4703	0.5562	0.8748
	**Mean**	**0.0343**	**0.8531**	**4.7907**	**3.6463**	**Mean**	**1.0168**	**0.4449**	**4.1160**	**1.3313**	**Mean**	**2.4577**	**0.4636**	**0.4431**	**1.4343**
	**SD**	**0.0242**	**0.4696**	**2.6994**	**2.8823**	**SD**	**0.3269**	**0.0956**	**3.5607**	**0.2867**	**SD**	**2.5893**	**0.0662**	**0.0885**	**0.4519**
White	WH(WL07)	0.0011	0.2627	0.6045	0.8163	WH(WL07)	0.2648	0.2217	0.0492	2.3829	WH(WL07)	2.9606	0.2480	0.9543	1.7067
	WH(WL07)	0.0011	0.2627	0.6045	0.8163	WH(WL07)	0.2648	0.2217	0.0492	2.3829	WH(WL07)	2.9606	0.2480	0.9543	1.7067
	WH(WL07)	0.0011	0.2627	0.6045	0.8163	WH(WL07)	0.2648	0.2217	0.0492	2.3829	WH(WL07)	2.9606	0.2480	0.9543	1.7067
	WH(WL19)	0.0593	0.2900	0.0074	1.5392	WH(WL15)	1.2576	0.6098	0.0155	1.7163	WH(WL19)	1.1397	0.6089	0.8214	0.6345
	WH(WL19)	0.0593	0.2900	0.0074	1.5392	WH(WL15)	1.2576	0.6098	0.0155	1.7163	WH(WL19)	1.1397	0.6089	0.8214	0.6345
	WH(WL19)	0.0593	0.2900	0.0074	1.5392	WH(WL15)	1.2576	0.6098	0.0155	1.7163	WH(WL19)	1.1397	0.6089	0.8214	0.6345
	WH(WL10)	0.7142	0.2927	0.0773	1.0374	WH(WL19)	0.3806	0.2031	0.0041	0.9357	WH(WL10)	1.0062	0.1924	0.5435	1.5550
	WH(WL10)	0.7142	0.2927	0.0773	1.0374	WH(WL19)	0.3806	0.2031	0.0041	0.9357	WH(WL10)	1.0062	0.1924	0.5435	1.5550
	WH(WL10)	0.7142	0.2927	0.0773	1.0374	WH(WL19)	0.3806	0.2031	0.0041	0.9357	WH(WL10)	1.0062	0.1924	0.5435	1.5550
	**Mean**	**0.2582**	**0.2818**	**0.2298**	**1.1310**	**Mean**	**0.6343**	**0.3449**	**0.0229**	**1.6783**	**Mean**	**1.7022**	**0.3498**	**0.7731**	**1.2987**
	**SD**	**0.3429**	**0.0144**	**0.2827**	**0.3208**	**SD**	**0.4701**	**0.1989**	**0.0203**	**0.6273**	**SD**	**0.9456**	**0.1958**	**0.1816**	**0.5025**
Student's *t*-test		0.4554	0.2969	0.0041	0.1275		0.1054	0.9782	0.0202	0.8720		0.9840	0.1868	0.0622	0.4053

Student's *t*-test was performed to test the significance of the RQ values of the oocyan samples relative to those of the non-oocyan (brown and white) samples. Significant *t*-test results ($P < 0.05$) are highlighted in yellow.
