# Supplementary material for: Endogenous Retrovirus EAV-HP Linked to Blue Egg Phenotype in Mapuche Fowl
Source: PLoS One. 2013 Aug 19;8(8):e71393. doi: 10.1371/journal.pone.0071393 (PMC3747184; doi:10.1371/journal.pone.0071393)
Supplement: Table S3 — Summary of samples used in the study. (PDF) [file pone.0071393.s005.pdf]

Supplementary Table S3. Summary of samples used in the study

Sample	Country[1]	*N*	Target-enrichment	Sanger-sequenced	qRT-PCR	*O*[2]	Source[3]	Extraction[4]
Araucana	GBR	1	ARA1	ARA1		*O*	PB	T
Araucana	FRA	5	FR2424			*O*	MTB	B
Araucana	FRA	4			OB25, OV14, OV5, OV8	*O*	MTB	T
Mapuche fowl	CHL	23	CH04, CH11, CH19, CH21			o^+/o^+	JAA	F
Mapuche fowl	CHL	31	CH20	CH20		*O*	JAA	F
Cream Legbar	GBR	2				*O*	PB	T
Crevecoeur	GBR	1	CRE10			o^+/o^+	PB	T
Dongxiang	CHN	51				?	HJ	F
Dongxiang	CHN	1		LK23[5]		*O*	HJ	F
Gallus gallus		2				o^+/o^+	HJ	B
Gallus gallus bankiva		2				o^+/o^+	HJ	B
Gallus gallus gallus		2				o^+/o^+	FRH	F
Gallus gallus spadiceus		2				o^+/o^+	HJ	B
Gallus lafayettii	NLD	2				o^+/o^+	FRH	F
Gallus sonneratii	NLD	2				o^+/o^+	FRH	F
Gallus varius	NLD	2				o^+/o^+	FRH	F
Moss	ESP	2				*O*	AMB	F
Rhode Island Red	FRA	4			OB18, OB21, OB26, OV10	o^+/o^+	MTB	T
White Leghorn	FRA	4			WL10, WL15, WL19, WL7	o^+/o^+	MTB	T
White Star	GBR	1	WST1T1			o^+/o^+	PB	T

[1] Sample origin, three-letter country code (ISO 3166-1 alpha-3)

[2] Oocyan phenotype: Blue/green eggs (*O*), non-blue/green eggs (o^+/o^+)

[3] Private Breeders (PB), Michele Tixier-Boichard (MTB), Jose Antonio Alcalde (JAA), Fazanterie de Rooie Hoeve (FRH), Ana Martinez-Bas (AMB), Han Jianlin (HJ)

[4] DNA extraction method: Tissue (T), Blood (B), FTA card (F)

[5] Dongxiang sample selected for sequencing based on genotyping from multiplex PCR
