# Supplementary material for: Endogenous Retrovirus EAV-HP Linked to Blue Egg Phenotype in Mapuche Fowl
Source: PLoS One. 2013 Aug 19;8(8):e71393. doi: 10.1371/journal.pone.0071393 (PMC3747184; doi:10.1371/journal.pone.0071393)
Supplement: Table S4 — Primers used in qRT-PCR. (PDF) [file pone.0071393.s006.pdf]

Supplementary Table S4. Primers used in qRT-PCR

QRT-PCR target	Primer sequence	GenBank Accession No.[1]
HMOX1-F	5'-GTGCACGAGCAGGCGGAGAA-3'	NM_205344.1
HMOX1-R	5'-ACGCCGTGACCAGCTTGAACT-3'	NM_205344.1
PDE3A-F	5'-TTGATGCCAGGAAAATGGAT-3'	XM_416416.3
PDE3A-R	5'-CCTTCTCCTCGTCCTCTTCC-3'	XM_416416.3
SLCO1B3-F	5'-TGGTGATTGCATTTGTAAGCTA-3'	XM_416418.3
SLCO1B3-R	5'-TGGAGAGCAGGGATTTATGC-3'	XM_416418.3
SLCO1C1-F	5'-ACCATGGGACCAAGCAGAAGGT-3'	NM_001039097.1
SLCO1C1-R	5'-CAGCCAGCGAGACACGCAGA-3'	NM_001039097.1
UB-F	5'-GGGATGCAGATCTTCGTGAAA-3'	X02650.1
UB-R	5'-CTTGCCAGCAAAGATCAACCTT-3'	X02650.1

[1] GenBank Accession No relates to the transcript used for the design of the primers.
