# Supplementary material for: Endogenous Retrovirus EAV-HP Linked to Blue Egg Phenotype in Mapuche Fowl
Source: PLoS One. 2013 Aug 19;8(8):e71393. doi: 10.1371/journal.pone.0071393 (PMC3747184; doi:10.1371/journal.pone.0071393)
Supplement: Table S5 — Primers used in long-range and multiplex PCRs. (PDF) [file pone.0071393.s007.pdf]

Supplementary Table S5. Primers used in long-range and multiplex PCR

**Primer**	**Primer sequence**	**Reference**
Long-range PCR-F	5'-AGTCAGGACACCCCATTTTT-3'	galGal3
Long-range PCR-R	5'-GGTTCTGTATCCACAACAACAGA-3'	galGal3
Primer walk 1F	5'-CGCTGAGATTGAAGCAACAC-3'	
Primer walk 1R	5'-GGAGATCGCGACAGTTTTGT-3'	
Primer walk 2F	5'-TTCCCCTTTTTGAACCACAC-3'	
Primer walk 2R	5'-GGGGTAGCAAGGATGATTGA-3'	
Multiplex PCR F	5'-GCATTTCACAAACGGGTGTA-3'	galGal3
Multiplex PCR R1	5'-CAAAACCACAAAGGTAATGTTCA-3'	galGal3
Multiplex PCR R2	5'-CCCAGCAGTAAGCCCTACAT-3'	EAV-HP (NC_005947.1)
