# Supplementary material for: Endogenous Retrovirus EAV-HP Linked to Blue Egg Phenotype in Mapuche Fowl
Source: PLoS One. 2013 Aug 19;8(8):e71393. doi: 10.1371/journal.pone.0071393 (PMC3747184; doi:10.1371/journal.pone.0071393)
Supplement: Methods S1 — Supplementary information on (a) sampling, (b) structural variant discovery, and (c) PCR cycling profiles. (PDF) [file pone.0071393.s008.pdf]

Supplementary Methods S1

(a) Sampling

DNA was isolated using in-house protocols depending on the type of tissue and method of collection/preservation. This included extraction from tissue, blood, and blood preserved on FTA Classic cards (Whatman®, GE Health Care). The samples used for RNA isolation were collected from a population of chickens maintained at the Institut National de la Recherche Agronomique (INRA), France. This population includes White Leghorn and Rhode Island Red breeds in which oocyan was introduced by cross-breeding with Araucana chickens in the 1970s. Oviposition time was recorded over 10 days for a subset of one year old 50 laying hens from the three strains. The subject hens were housed individually for accurate recording of laying performance. Blood and tissue sampling took place at the end of the observation period 1 to 3 hours before expected time of lay. The location of the egg in the reproductive tract was recorded post-slaughter, together with its condition – whether calcified or soft-shelled. Tissues were sampled from the oviduct, shell gland and liver, and stored in Nalgen cryotubes with 2 ml RNA-*later*® Solution (Life Technologies Ltd). The tissues were shipped to the UK where they were stored at -80°C.

(b) Structural variant discovery

Structural variants (SVs) were called using GASV 2.0 [1] after preparation of BAM files using the BAM preprocessor Perl script provided with GASV 1.5. The BAM preprocessor v2.0 requires that both reads mapping in a pair pass the minimum quality value (QV) setting, whilst v1.5 requires that only one of the reads mapping in a pair passes the QV setting. GASV reports on SV events defined by clusters of discordantly mapped fragments. Predicted SVs include deletions, inversions, translocations, and divergent events – the latter being an inter-chromosomal rearrangement that is not a deletion, inversion or insertion. Non-default settings for GASV included a read length of 100, -lmin 200, -lmax 400, a minimum cluster size of 4, and an increase in the number of chromosomes

to 33 (1-28, 32, Z, W, M and UnRandom). SV analysis was performed on oocyan and non—oocyan chickens independently, and subsequently filtered to produce a set of SVs unique to oocyan. SVs were then filtered by chromosome and position to retain only those within the mapping interval (Gga1:67,051,487-67,364,512), and supported by a minimum of 12 paired-end sequences (PES); 12 equated to the minimum GASV cluster size (size = 4), multiplied by the number of oocyan samples (n = 3). PES spanning the SV boundaries on the 'left' chromosome +/- 200 bp were extracted using SAMtools [2], converted to FASTA format and assembled with CAP3 [3]. A BLAT search of the CAP3 contigs to the chicken genome (galGal3) was performed to identify sub-sequences that mapped to the 'right' chromosome and boundaries of the candidate SV. Where the BLAT search successfully aligned contigs to both the 'left' and 'right' chromosomes and boundaries it allowed the break-point of a candidate SV to be resolved at base-pair resolution. Clustal [4] alignment of contig sub-sequences mapping to the 'left' and 'right' chromosomal insertion provided two sequences (Seq1 and Seq2) representing the tail ends of a sequence insertion.

(c) PCR cycling profiles

qRT-PCR was performed as follows: 50 ng/µl of the cDNA was used as the template in a quantitation comparative ($\Delta\Delta C_T$) qRT-PCR in a 10 µl reaction mix containing 1X SYBR green chemistry (PrimerDesign® Ltd) and 200 nM of each primer pair on 96-well reaction plates (Applied Biosystems). qRT-PCR was performed using Applied Biosystems 7500 Fast Real-Time PCR Systems v2.0.6 (Life Technologies), and the reaction profile involved an initial holding stage at 95°C for 3 min followed by 40 cycles of denaturation at 95°C for 30s and a combined primer annealing/elongation step at 61°C for 30s. This cycle was followed by a melting curve analysis step involving a temperature range from 95°C for 15s to 61°C for 1 min. Except for the blank controls that were run in a single replicate for each target, three replicate reactions were performed for each tissue and gene target.

Long-range touch-down PCR was performed as follows: 1 µl of 100 ng/µl template was used in PCR reaction mix having 4 µl 5X HF Buffer (New England BioLabs (UK) Ltd), 0.2 mM dNTP, 0.4 µM each primer, and 0.2 µl *Taq* (Phusion® Polymerase; New England Biolabs (UK) Ltd), made to a final volume of 20 µl with nuclease-free water. The PCR cycling profile involved an initial denaturation at 98ºC for 30s; followed by 6 cycles of 98ºC for 30s, the touch-down temperature (which started at 72ºC and decreased to 66ºC over the course of the 6 cycles) for 30s, and extension at 72ºC for 2 min 30s. These were followed by 25 cycles of 98ºC for 30s, 66ºC for 30s, 72ºC for 2 min 30s; and a final extension of 72ºC for 5 min. PCR products were analyzed by 0.8% agarose gel electrophoresis using a 2-Log DNA ladder (New England BioLabs (UK) Ltd).

Multiplex PCR was performed as follows: 1 µl of 20 ng/µl template was used in PCR reaction mix having 4 µl 5X HF Buffer (New England BioLabs (UK) Ltd), 0.2 mM dNTP, 0.4 µM each primer, 0.2 µl *Taq* (Phusion® Polymerase, New England Biolabs (UK) Ltd), made to a final volume of 20 µl with 13.8 µl nuclease-free water. The PCR cycling profile involved an initial denaturation at 98ºC for 30s; followed by 35 cycles of 98ºC for 10s, 66ºC for 30s, 72ºC for 30s; and a final extension step at 72ºC for 5 min. PCR products were analyzed by 3.5% agarose gel electrophoresis using HyperLadder V (Bioline Reagents Ltd).

References

1. Sindi S, Helman E, Bashir A, Raphael BJ (2009) A geometric approach for classification and comparison of structural variants. Bioinformatics 25: i222–i230. doi:10.1093/bioinformatics/btp208.

2. Li H, Handsaker B, Wysoker A, Fennell T, Ruan J, et al. (2009) The Sequence Alignment/Map format and SAMtools. Bioinforma Oxf Engl 25: 2078–2079. doi:10.1093/bioinformatics/btp352.

3. Huang X, Madan A (1999) CAP3: A DNA sequence assembly program. Genome Res 9: 868–877.

4. Larkin MA, Blackshields G, Brown NP, Chenna R, McGettigan PA, et al. (2007) Clustal W and Clustal X version 2.0. Bioinforma Oxf Engl 23: 2947–2948. doi:10.1093/bioinformatics/btm404.
