# Supplementary figures and images for: Endogenous Retrovirus EAV-HP Linked to Blue Egg Phenotype in Mapuche Fowl
Source: PLoS One. 2013 Aug 19;8(8):e71393. doi: 10.1371/journal.pone.0071393 (PMC3747184; doi:10.1371/journal.pone.0071393)

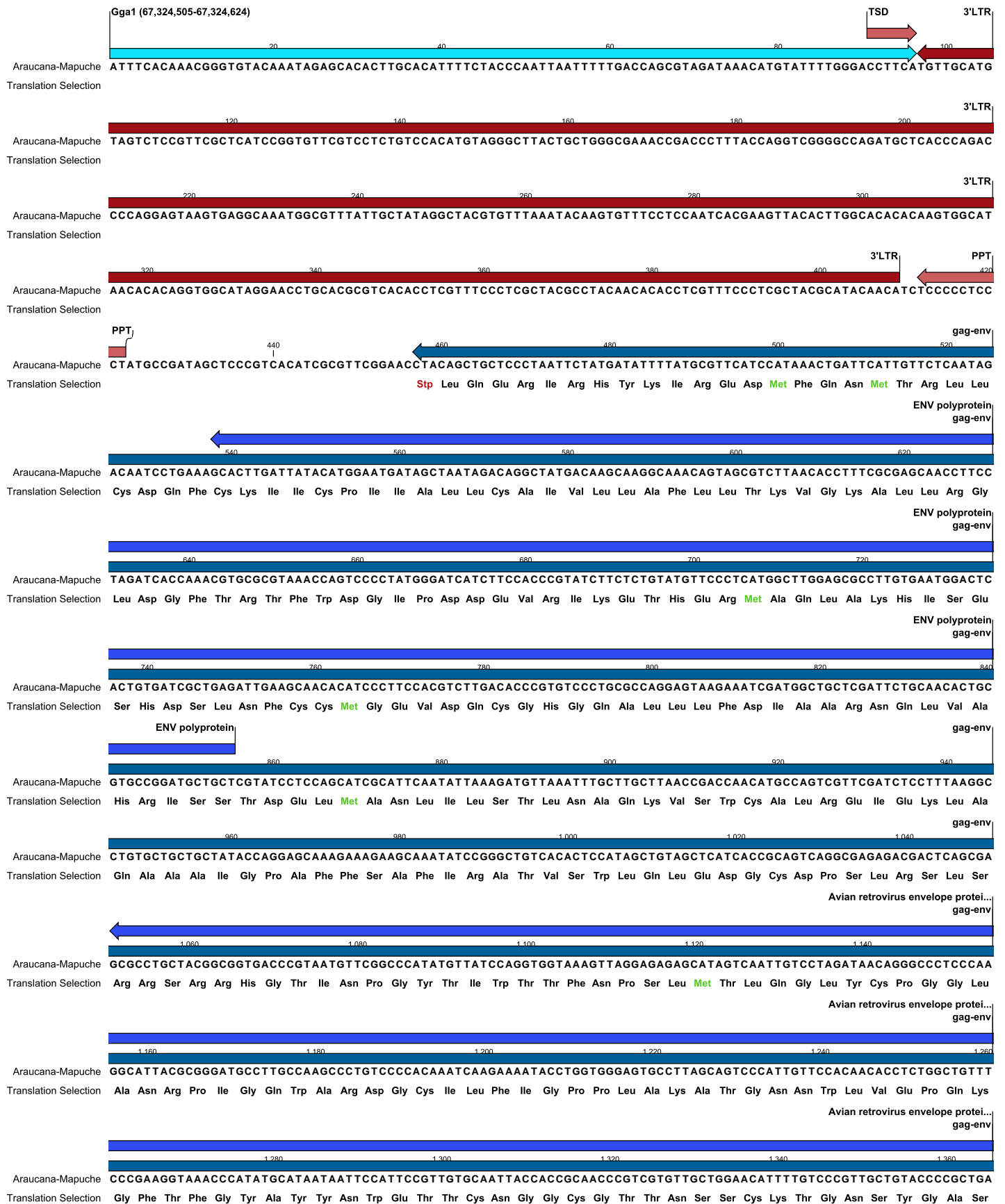



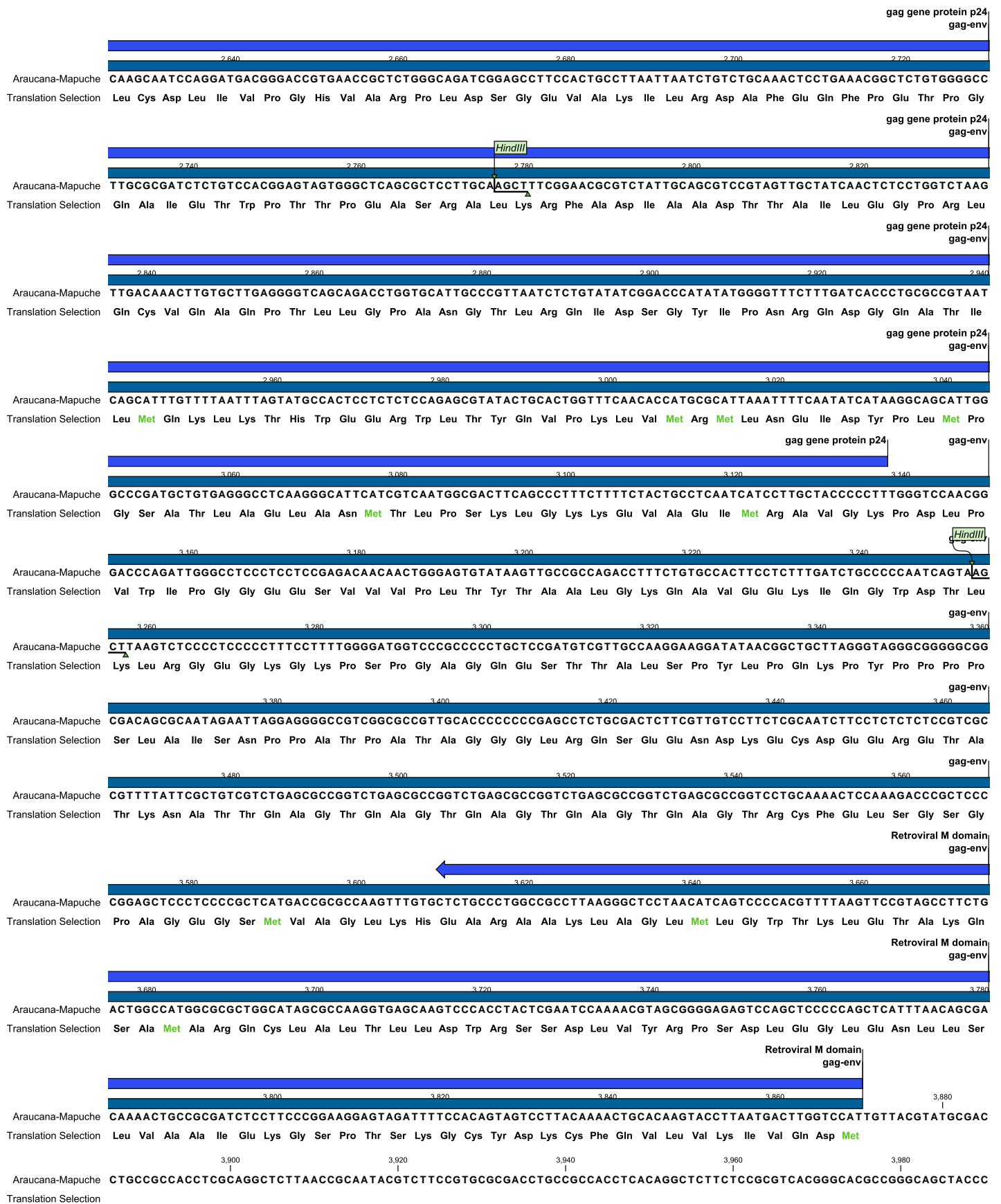

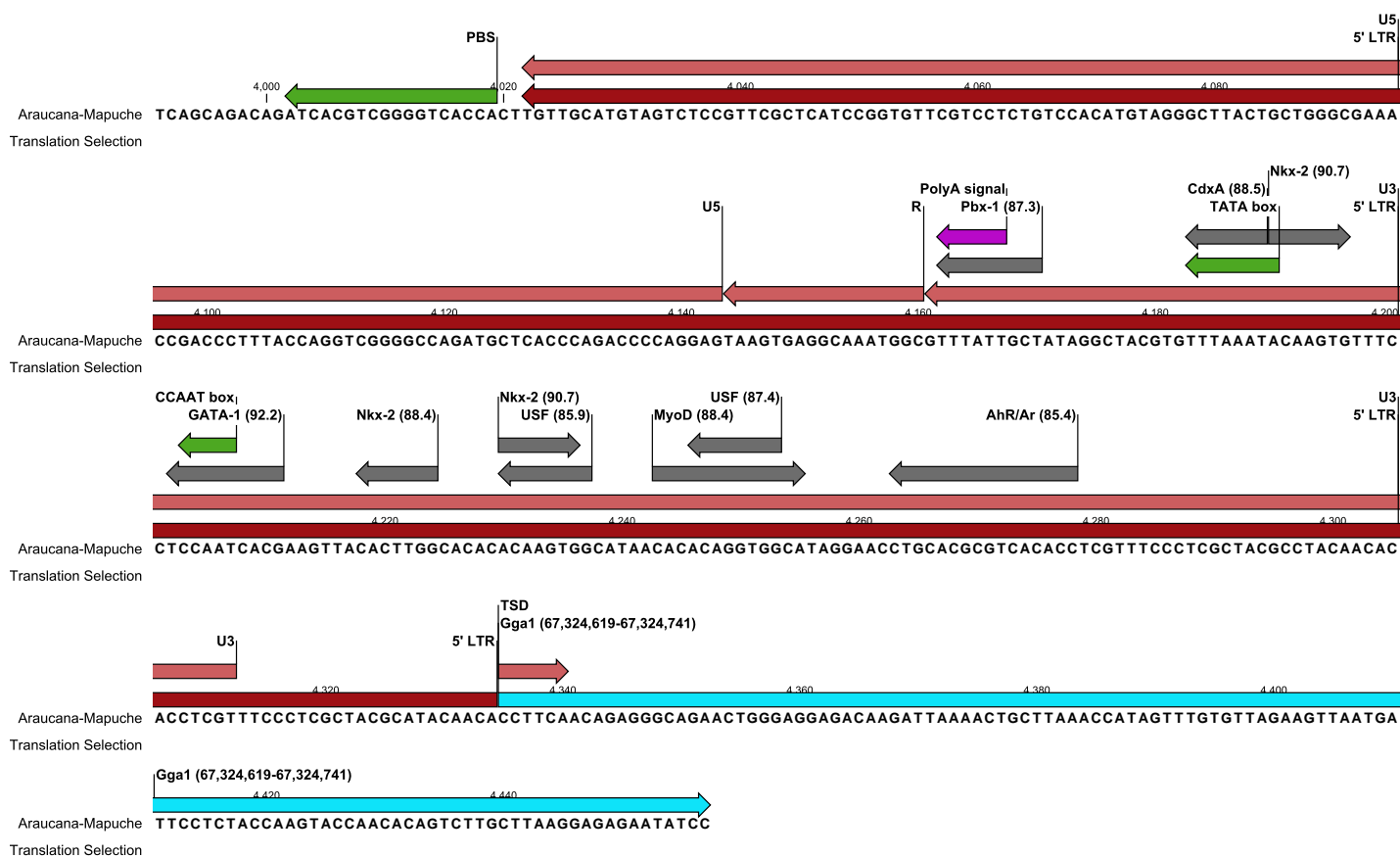

Supplement: Sequence S1 — Annotated sequence for Mapuche/Araucana oocyan homozygote. (PDF) [file pone.0071393.s009.pdf]

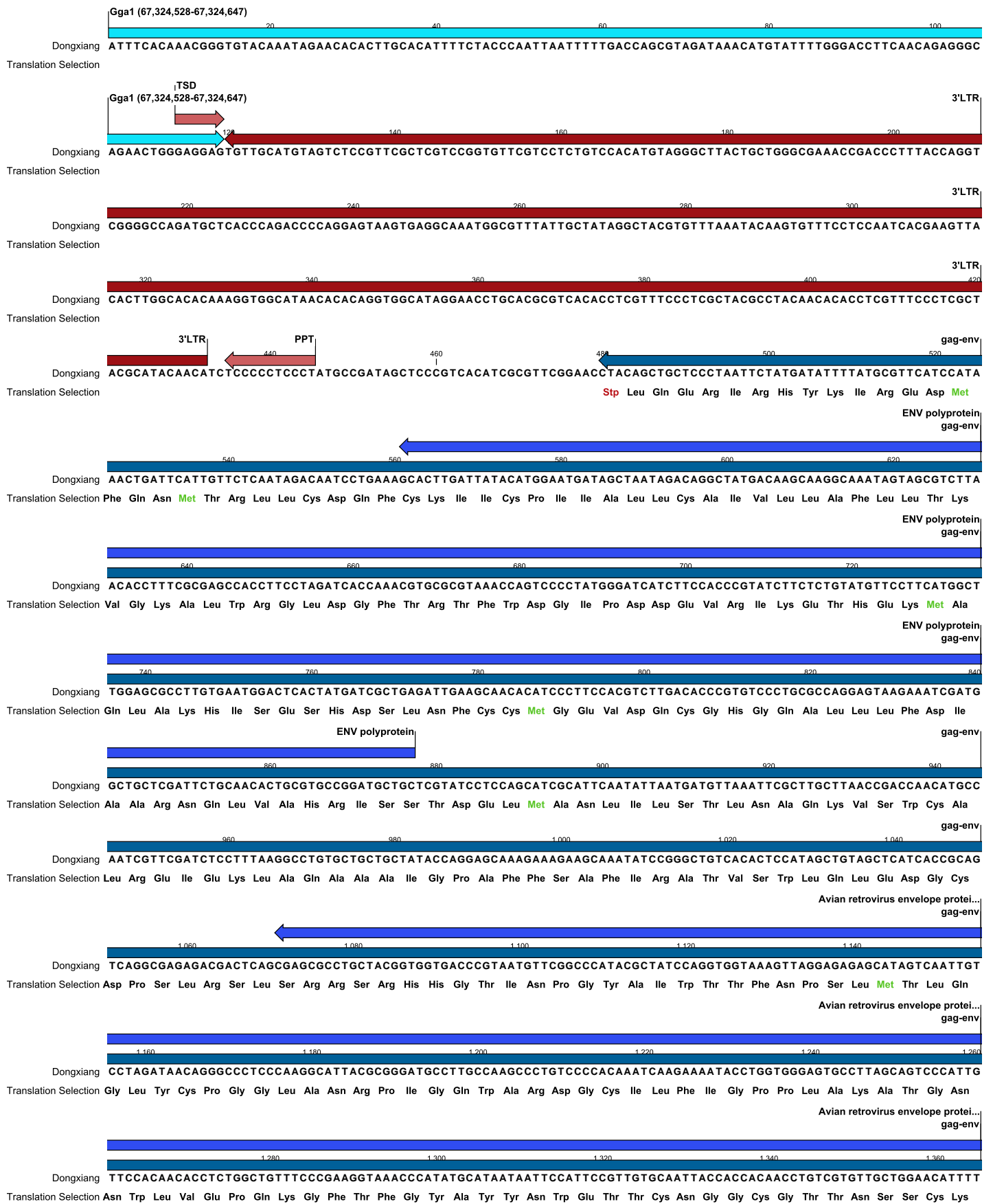

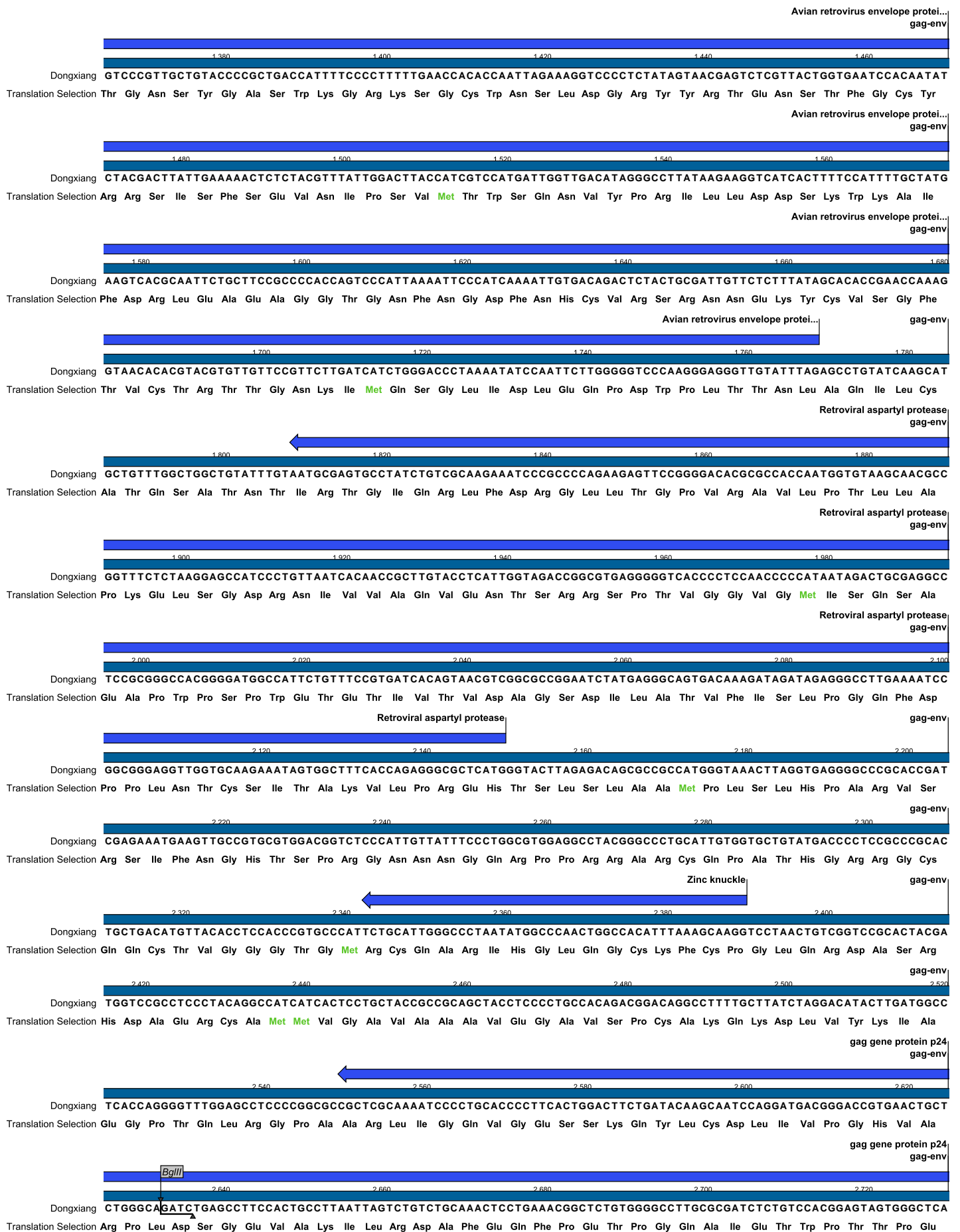

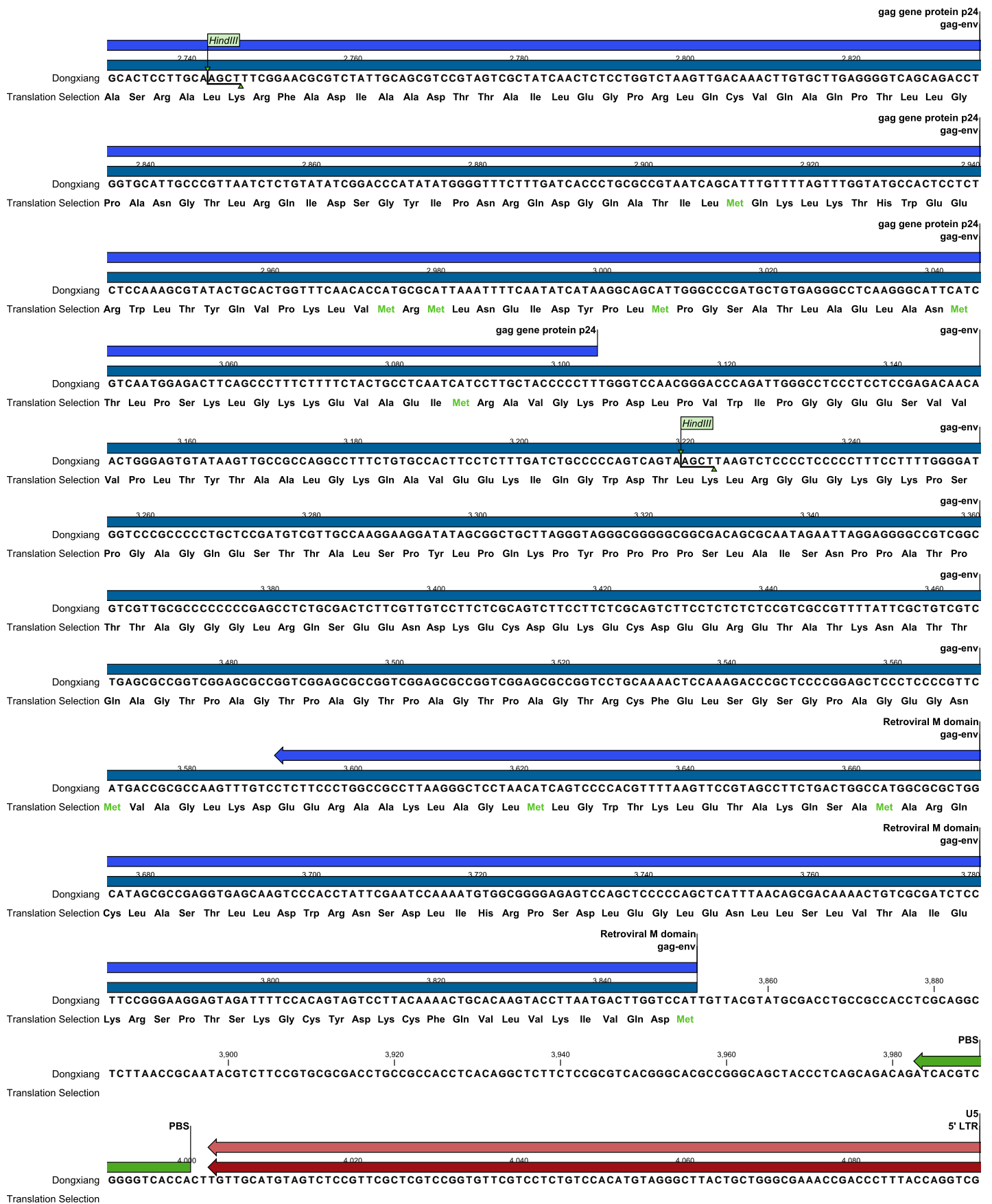

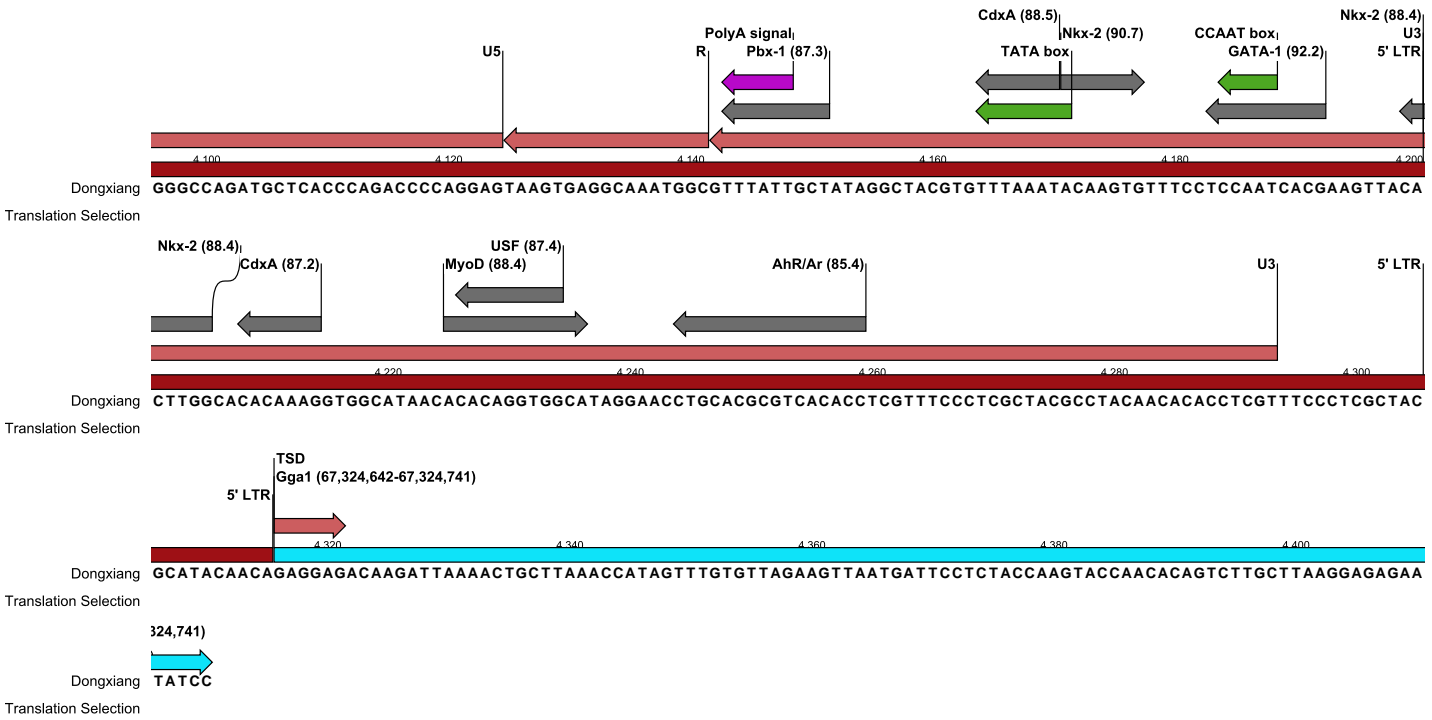

Supplement: Sequence S2 — Annotated sequence for Dongxiang oocyan homozygote. (PDF) [file pone.0071393.s010.pdf]

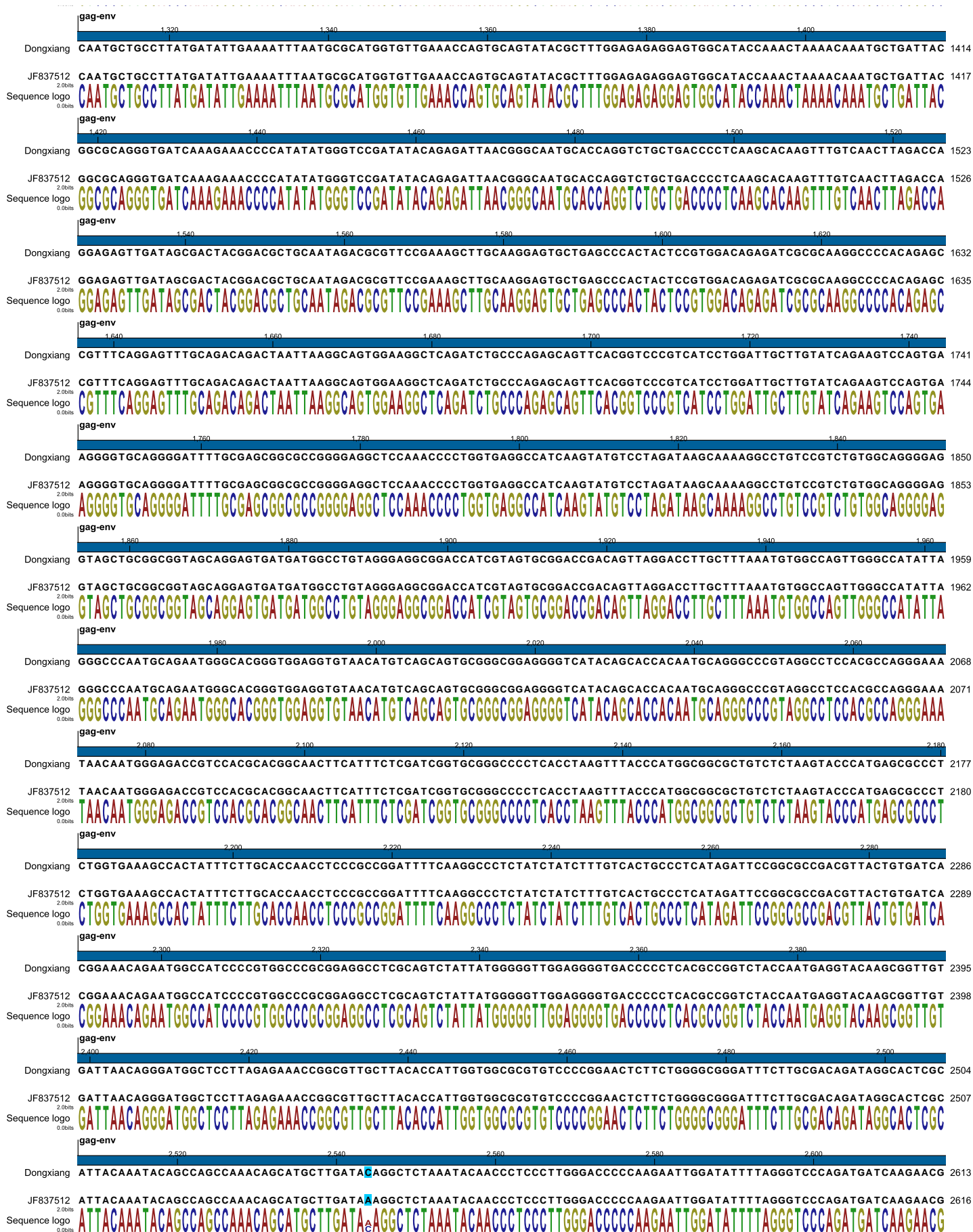

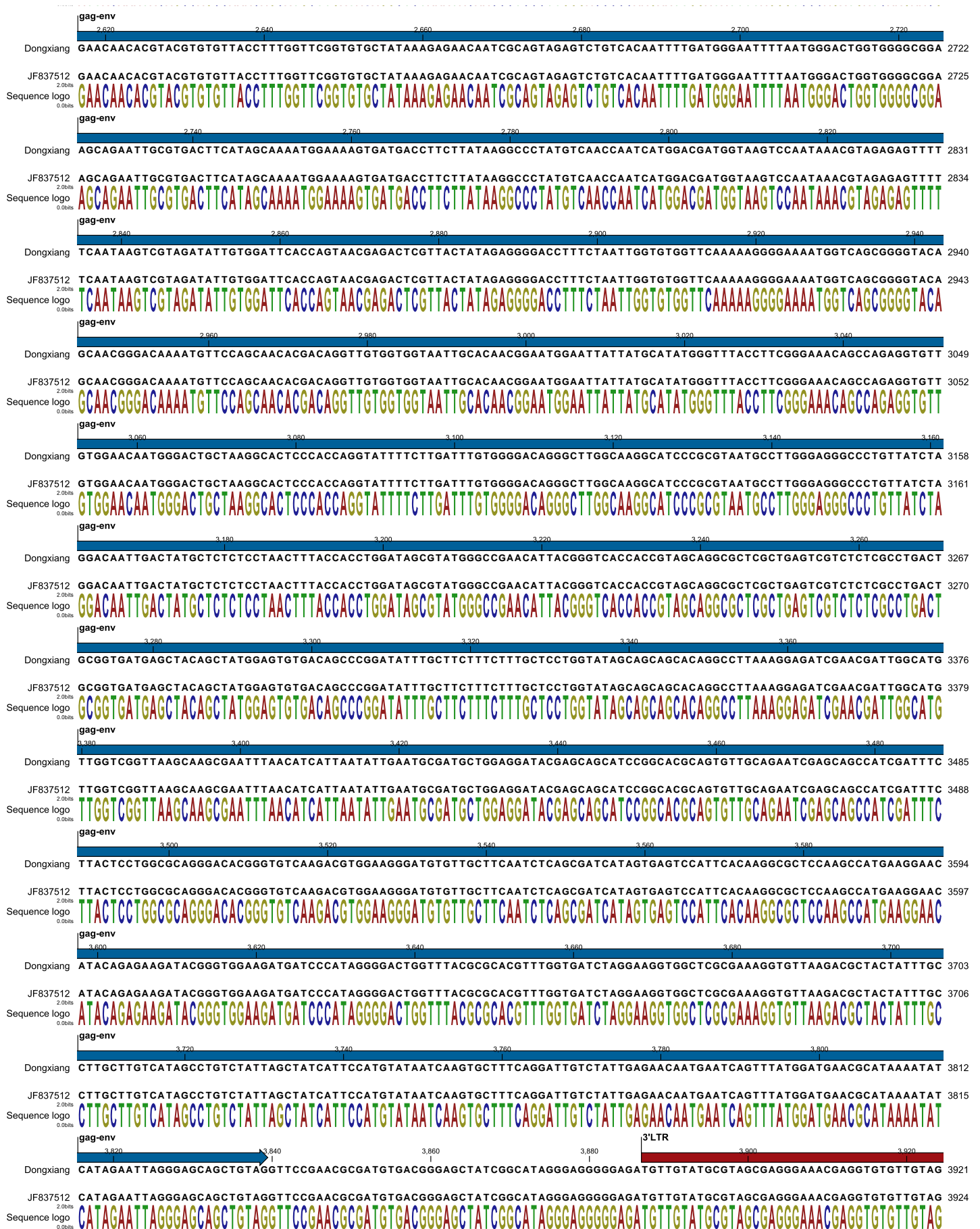

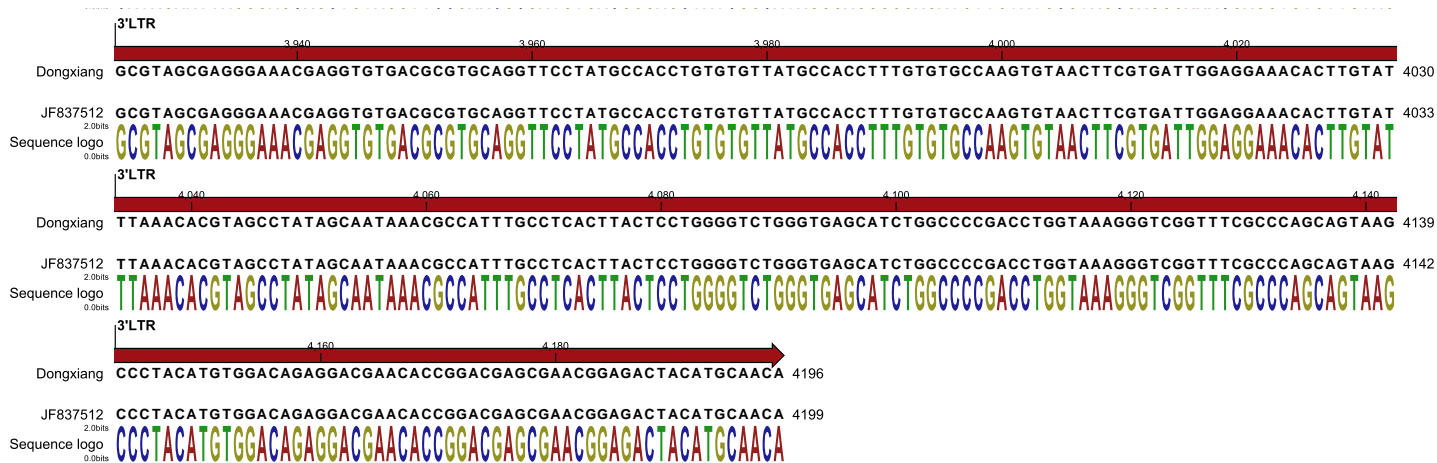

Supplement: Sequence S3 — Sequence alignment of Dongxiang sequence to GenBank accession no: JF837512. (PDF) [file pone.0071393.s011.pdf]
